# Population pharmacokinetics of buprenorphine and naloxone sublingual combination in Chinese healthy volunteers and patients with opioid use disorder: Model-based dose optimization

**DOI:** 10.3389/fphar.2023.1089862

**Published:** 2023-01-19

**Authors:** Meng Gu, Anning Li, Wenyao Mak, Fang Dong, Nuo Xu, Jingye Zhang, Yufei Shi, Nan Zheng, Zhijia Tang, Qingfeng He, Canjun Ruan, Wei Guo, Xiaoqiang Xiang, Chuanyue Wang, Bing Han, Xiao Zhu

**Affiliations:** ^1^ Department of Clinical Pharmacy and Pharmacy Administration, School of Pharmacy, Fudan University, Shanghai, China; ^2^ Department of Pharmacy, Minhang Hospital, Fudan University, Shanghai, China; ^3^ The National Clinical Research Center for Mental Disorders & Beijing Key Laboratory of Mental Disorders, Beijing Anding Hospital, Capital Medical University, Beijing, China; ^4^ Advanced Innovation Center for Human Brain Protection, Capital Medical University, Beijing, China

**Keywords:** buprenorphine + naloxone, sublingual, population pharmacokinetics, parent-metabolite model, opioid use disorder

## Abstract

The sublingual combination of buprenorphine (BUP) and naloxone (NLX) is a new treatment option for opioid use disorder (OUD) and is effective in preventing drug abuse. This study aimed to explore rational dosing regimen for OUD patients in China *via* a model-based dose optimization approach. BUP, norbuprenorphine (norBUP), and NLX plasma concentrations of 34 healthy volunteers and 12 OUD subjects after single or repeated dosing were included. A parent-metabolite population pharmacokinetics (popPK) model with transit compartments for absorption was implemented to describe the pharmacokinetic profile of BUP-norBUP. In addition, NLX concentrations were well captured by a one-compartment popPK model. Covariate analysis showed that every additional swallow after the administration within the observed range (0–12) resulted in a 3.5% reduction in BUP bioavailability. This provides a possible reason for the less-than-dose proportionality of BUP. There were no differences in the pharmacokinetic characteristics between BUP or NLX in healthy volunteers and OUD subjects. Ethnic sensitivity analysis demonstrated that the dose-normalized peak concentration and area-under-the-curve of BUP in Chinese were about half of Puerto Ricans, which was consistent with a higher clearance observed in Chinese (166 L/h vs. 270 L/h ). Furthermore, Monte Carlo simulations showed that an 8 mg three-times daily dose was the optimized regimen for Chinese OUD subjects. This regimen ensured that opioid receptor occupancy remained at a maximum (70%) in more than 95% of subjects, at the same time, with NLX plasma concentrations below the withdrawal reaction threshold (4.6 ng/mL ).

## 1 Introduction

Opioid abuse can lead to adverse consequences and death. According to the 2019 report published by the International Drug Policy Consortium (IDPC), opioid abuse in China was serious with 850,000 opioid use disorders (OUD) subjects in the country. Buprenorphine (BUP) is one of the agonistic substitution drugs commonly used to treat OUD. It has a high μ-opioid receptor ( μOR ) affinity and can relieve the withdrawal reaction of OUD by partially agonizing μOR . Although BUP is less likely to cause dependence than the full agonist methadone, BUP is still frequently abused and this remains a serious problem worldwide. Meanwhile, Naloxone (NLX) is a full μOR antagonist that causes a strong withdrawal response when administered intravenously but has low oral bioavailability. Hence, BUP and NLX sublingual compound, such as the marketed SUBOXONE^®^, is an effective way to minimize the abuse of BUP.

Although sublingual administration circumvents first-pass metabolism, the pharmacokinetics (PK) of BUP exhibits a less-than-dose proportionality profile, especially at high doses (Chiang and Hawks, 2003). It has been hypothesized that increased swallowing by OUD subjects during high dose administration is responsible for the decreased bioavailability, which increases the proportion entering the gastrointestinal tract. Currently there is few prospective studies that examined this phenomenon. Clinical studies have suggested that BUP plasma concentration is positively associated with μ-opioid receptor occupancy ( μORO ) which reflects its efficacy (Greenwald et al., 2003; Greenwald et al., 2007). As such, a quantitative description of the relationship between concentration and dose of BUP, as well as the impacts of covariates such as weight and race, is essential for dose adjustment and efficacy assessment.

In 2006, Ashraf Yassen et al. (2006) established a population pharmacokinetic/pharmacodynamic (popPK/PD) model of BUP injection, characterizing for the first time the PK behavior of BUP in healthy volunteers. Recently, Darlene Santiago et al. (2020) used a two-compartment model to characterize the PK profile of sublingual BUP tablets in Puerto Rican OUD subjects. However, there are limited popPK models of sublingual BUP tablets, most of which had small sample sizes and limited the robustness results, especially in OUD subjects. To our knowledge, no popPK studies of BUP and NLX in BUP/NLX formulations existed at the time of writing. As NLX has a low sublingual bioavailability, its clinical use should result in a low plasma concentration that does not exceed the withdrawal threshold, but studies that ascertain the NLX plasma concentration in Chinese subjects were still lacking (Culpepper-Morgan et al., 1992). Furthermore, there is only one non-compartmental analysis (NCA) study of BUP/NLX formulation for the Chinese population, and population pharmacokinetics studies were lacking (Dong et al., 2019). Therefore, there is an urgent need to develop a popPK model of BUP/NLX sublingual tablets for dose optimization of OUD treatment in Chinese.

The overarching aim of this study was to determine the population pharmacokinetics of BUP/NLX sublingual tablets in the Chinese population to guide rational dosing. Specifically, we aimed to: 1) Perform a non-compartmental analysis and evaluate the impact of ethnic difference on the PK of BUP; 2) develop a popPK model for BUP/NLX sublingual tablets and assess the effect of swallowing on the bioavailability of BUP; 3) perform model-based dose optimization by balancing the efficacy and toxicity of the treatment.

## 2 Materials and methods

### 2.1 Study design

Data used for analyses were extracted from a clinical trial that utilized both the single and multiple dosage regimens of BUP and NLX (each tablet contains 2 mg buprenorphine hydrochloride and 0.5 mg naloxone hydrochloride), where plasma concentrations were measured in eligible subjects. The trial was conducted in accordance with Chinese legal requirements and the Declaration of Helsinki, and was approved by the Ethics Committee of Beijing Anding Hospital, China (approval number: 2008L10448).

### 2.2 Subjects

This study included single and multiple dosing trials. Healthy volunteers were recruited as the single dosing regimen should not lead to addiction. On the other hand, considering the addiction risk of the multiple dosing regimens, subjects in the multiple dosing group were recruited from a rehabilitation center after informed consent had been given freely. Inclusion criteria included subject aged between 18–30 years and 18–45 years respectively in the single- and multiple-dose group, and should weighed no less than 50 kg. Exclusion criteria included a history of cardiac, hepatic, renal, respiratory, gastrointestinal, or neurological disease, and a history of psychiatric or metabolic disease. Healthy volunteers had not taken other drugs within 2 weeks before the clinical trial, and OUD subjects were not allowed to take other drugs during the trial period. In addition, physical examination and urine morphine concentration of OUD subjects should not be abnormal. Subjects who had missed a dose, or received a wrong dose (either more or less than the prescribed dose) during the trial were also excluded from the analysis. All subjects provided informed consent prior to any study-related activity.

### 2.3 Dosing scheme

The single-dose trial consisted of three dose groups, 4 mg, 8 mg, and 16 mg, which were administered at 8:00 a.m. on the first day of the study. The multiple-dose trial only had one dose group, and subjects were given a daily dose at 8:00 a.m. consecutively for 8 days. All doses were administered sublingually. To protect healthy subjects, naltrexone was given at 6 p.m. the day before the study, at 7 a.m. on the first day, and at 8 a.m. on the second day. Subjects in the 4 mg, 8 mg, and 16 mg groups were given naltrexone 25 mg, 50 mg, and 100 mg, respectively, to avoid the risk of physical dependence.

### 2.4 Blood and number of swallows sampling

In the single-dose trial, the blood concentrations of BUP, norBUP, and NLX were collected before dose administration and at 0.25, 0.5, 0.75, 1, 1.5, 2, 3, 4, 6, 8, 10, 12, 24, 36, 48, 60, and 72 h after administration. Blood samples from the multiple-dose trial were collected on the sixth, seventh, and eighth day before administration, and 0.25, 0.5, 1, 1.5, 2, 4, 8, 12, and 24 h after administration on the eighth day only. In addition, the number of swallow (NOS) performed by the subject in the single-dose group after drug administration was recorded.

### 2.5 Analytical method

All collected samples were centrifuged within 1 h and stored at −20°C. The concentration of BUP, norBUP and NLX were quantified using a validated high-performance liquid chromatography-tandem mass spectroscopy (HPLC-MS) method. The mobile phase for BUP and norBUP detection was 80% methanol (0.005 mol/L ammonium format and 0.1% formic acid), and for NLX, the mobile phase was 36% acetonitrile (0.005 mol/L ammonium format and 0.1% formic acid). The internal standards for mass spectrometry were BUP-D4 (Lot No. 6-JHY-131-5, Toronto Research Chemicals), norBUP-D3 (Lot No. FE102710-01, Cerilliant) and naloxone-D5 (Lot No. FN081810-02, Cerilliant). The lower limit of quantification (LLOQ) of BUP and norBUP was 50 ng/L and that of NLX was 20 ng/L .

### 2.6 Non-compartmental pharmacokinetic analysis

Non-compartmental analysis was performed using R studio version 4.1.1 (ncappc package version 0.3.0). Pharmacokinetic parameters, including maximum concentration ( $C_{\max}$ ), time for maximum concentration ( $T_{\max}$ ) and area-under-the-curve (AUC) were calculated using the linear up-log-down method from individual concentration-time data for all subjects. To assess the ethnic difference between Chinese and Puerto Ricans, $T_{\max}$ , dose-normalized $C_{\max}$ and AUC of the 8 mg single dose group for 0–8 h were also calculated (Santiago et al., 2020).

## 3 Overview of population pharmacokinetic analysis

The non-linear mixed-effects modeling software NONMEM (version 7.5, ICON Development Solutions, MD, United States) was used to develop the popPK models. The evaluation of the NONMEM outputs was performed with Perl-speaks-NONMEM (PsN, version 5.2.6, Uppsala University, Sweden) and Pirana (version 3.0.0, Certara, United States) was used to handle modeling workflow. For statistical analysis and output visualization, R (version 4.1.1) was employed. Visual predictive check (VPC) and prediction corrected visual predictive check (pcVPC) were performed using the R package tidyvpc (version 1.2.0, Certara, United States). When the percentage of below quantification limit (BQL) data was less than 10%, BQL data were removed (M1 method) and the first-order conditional estimation with interaction (FOCE-I) method was used for parameter estimations. On the other hand, the likelihood-based method (M3 method) along with LAPLACIAN estimation was used if the proportion of BQL data was more than 10% or when necessary (Ahn et al., 2008). Since there was no pharmacokinetic interaction between BUP and NLX, PK models for BUP parent-metabolite model and NLX were developed separately (Harris et al., 2000). The parent-metabolite model was built using a sequential Population PK Parameters and Data (PPPD) approach (Zhang et al., 2003). First, the parent drug model was developed. Subsequently, the typical population parameters of the parent model were fixed and the plasma concentrations of both the parent drug and metabolite were fitted simultaneously to estimate the metabolite-related parameters. Finally, the parameters of the parent drug and the metabolite were estimated simultaneously based on the full data.

### 3.1 Base model development

#### 3.1.1 Buprenorphine parent-metabolite model

Based on published studies and exploratory analysis results (Supplementary Figure S1), one-, two- and three-compartment models were selected as candidate structural models for BUP. In addition, first order, first order with lag time and transit compartment model were tested for the absorption processes. As data on intravenous administration was lacking, it was challenging to accurately estimate the proportion of BUP metabolized to norBUP ( $F_{m}$ ). In this study, the proportion of BUP transformed to norBUP was assumed to be 100% and $F_{m}$ was fixed to 1 (Ahlers et al., 2015). Through the hysteresis loop plot (Supplementary Figure S4), it was found that there was no significant delay in the formation of norBUP. Therefore, the candidate structural model for norBUP is a one-, two-, and three-compartment model with first-order elimination (Supplementary Figure S2). The code of final model can be found in Supplementary Material.

#### 3.1.2 Naloxone model

Results from previous studies and exploratory analyses (Supplementary Figure S5) support one-, two- and three-compartment models with first-order absorption and first-order elimination as candidate structural models (Yassen et al., 2007; Dowling et al., 2008; Papathanasiou et al., 2019; Skulberg et al., 2019). The code of final model can be found in Supplementary Material.

### 3.2 Random effect model

The exponential model was used to model the inter-individual variability (IIV) of each pharmacokinetic parameter Eq. 1.

$$P_{i}=\theta \times \exp\left(\eta_{i}\right)$$ (1)

where θ is the typical value of the parameter and $\eta_{i}$ is the IIV term that is normally distributed with a mean of 0 and a variance of $\omega^{2}$ .

Specification of the residual error structure is evaluated, including additive, proportional, and combined error models. The equations are as follows Eqs. 2–4.

$$Y=IPRED+\varepsilon_{1}$$ (2)

$$Y=IPRED\times \left(1+\varepsilon_{2}\right)$$ (3)

$$Y=IPRED\times \left(1+\varepsilon_{2}\right){+\varepsilon }_{1}$$ (4)

Where Y and IPRED denote the measured concentration and individual prediction, respectively, $\varepsilon_{1}$ is the additive error and $\varepsilon_{2}$ is the proportional error, both of which are assumed to be Gaussian distributed with a mean of 0 and a variance of $\sigma_{1}^{2}$ and $\sigma_{2}^{2}$ , respectively.

The dosage form used in this clinical trial was a sublingual tablet, and had minimal absorption when swallowed (went into the gastrointestinal tract rather than the blood circulation). The bioavailability of BUP was poorer in the high-dose group and NOS was collected in the single-dose group (Chiang and Hawks, 2003). NOS was not recorded in the multiple-dose group due to operational challenges, but was imputed with the median from the single-dose 8 mg group (the same dose as the multiple-dose group). During modeling, NOS as an important covariate with clear physiological significance, was preferentially investigated in the BUP parent-metabolite structural model to examine its effects on absorption-related parameters (inclusion criterion was *p* < 0.01 and exclusion criterion *p* < 0.001).

Structural model selection was based on the value of Akaike information criterion (AIC), goodness-of-fit (GOF) plots, the precision of parameter estimations (as percentage relative standard error, % RSE), and the condition number.

### 3.3 Covariate analysis

The impact of covariates on PK parameters was evaluated. An exploratory analysis of all covariates was carried out by plotting. The correlation between covariates was investigated through exploratory data analysis and statistical evaluation. Analyses of variance (ANOVA) tests were performed for categorical covariates, while linear regression was used to analyze continuous covariates. For univariate analysis, a *p*-value of less than 0.05 (*p* < 0.05) was considered significant. Among the highly correlated covariates, the most biologically plausible covariates were selected for stepwise covariate modelling (SCM). SCM relies on likelihood ratio test (LRT) to automate the search for covariates and included both the forward inclusion and backward elimination steps. In forward inclusion, the covariates were retained when the addition of a covariate resulted in a reduction in objective function value (OFV) ≥ 6.64 (*p* < 0.01, df = 1). Backward elimination was performed after the forward inclusion was completed. If the deletion of a covariate increased OFV ≥ 10.83 (*p* < 0.001, df = 1), the covariate was retained. The final determination of covariates was decided based on statistical evidence and clinical knowledge. Continuous covariates were added into the model *via* a power or linear function, while discrete covariates were added *via* a linear function Eqs. 5–7.

$$P_{i}=\theta_{1}∙\left(1+\theta_{2}∙\left({Cov}_{con,\;i}-{Cov}_{median}\right)\right)$$ (5)

$$P_{i}=\theta_{1}∙{\left(\frac{{Cov}_{con,\;i}}{{Cov}_{median}}\right)}^{\theta_{2}}$$ (6)

$$P_{i}=\theta_{1}∙\left(1+\theta_{2}∙{Cov}_{cat,i}\right)$$ (7)

Where $\theta_{1}$ is the mean of the parameter in an individual with the covariance median value and $\theta_{2}$ represent the estimated typical value of the covariance effect on $\theta_{1}$ , ${Cov}_{con,\;i}$ is the $i^{th}$ individual’s continuous covariate value, ${Cov}_{cat,i}$ is the $i^{th}$ individual’s categorical covariate value, ${Cov}_{median}$ is the median of the continuous covariates, and $P_{i}$ is the parameter for the $i^{th}$ individual adjusted by covariates.

### 3.4 Model evaluation

The final model was graphically evaluated by GOF plots such as observed values (DV) *versus* individual prediction (IPRED) and population predictions (PRED), conditional weighted residuals (CWRES) *versus* time and PRED, individual weighted residual (IWRES) *versus* IPRED, and the normality test of CWRES. CWRES was replaced with normalized prediction distribution error (NPDE) in the GOF plots when the M3 method was used to handle BQL data.

Bootstrap was performed with PsN for internal validation of the model. One thousand sets of parameters were estimated by repeating 1,000 datasets from the original dataset after stratifying by different dose groups. Median values and 95% confidence intervals (CI) were derived and compared with the final model parameters to assess the robustness of the final model.

In addition, VPC, pcVPC and the numerical predictive check (NPC) were performed to assess the prediction power of the final model. One-thousand simulations were implemented and the 5th, 50th, and 95th percentiles of the observed and simulated data (included concentration, AUC and $C_{\max}$ ) were calculated. The results of VPC and pcVPC were presented graphically, and NPC showed the statistical results. In addition, the BQL data generated in the simulated dataset were used to test the appropriateness of the BQL data handling method in the final model, and the results were graphically represented as the fraction of BQL over time.

### 3.5 Dose optimization *via* Monte Carlo simulations

According to published data, suppression of opioid withdrawal responses requires plasma concentrations to be maintained above 1 ng/mL , which may require daily BUP dose of 4 mg. And 3 ng/mL or higher to obtain plasma BUP concentrations that inhibit the opioid reinforcing effects of typical doses of most illicit drugs, which may require daily BUP dose of 16 mg (Greenwald et al., 2014). Another study also showed that μORO reached a plateau with occupancy between 70% and 90% when BUP levels approached 2–3 ng/mL (Nasser et al., 2014). Therefore, 3 ng/mL appeared to be the threshold to ensure that BUP effectively treats OUD. Based on these studies, the dosing regimens used for simulation included 16 mg QD, 8 mg BID, 16 mg + 8 mg per day, 8 mg TID, 16 mg BID and 16 mg + 8 mg + 8 mg per day. Monte Carlo simulations were performed based on the established model in RxODE (version 1.1.2).

#### 3.5.1 Simulation of minimum suppression concentration

The minimum suppression concentration (MSC) was used to represent the threshold (3 ng/mL ) of BUP for the treatment of OUD, and the fraction of time with plasma concentration above MSC (%T > MSC) was used as an alternative PD index. Monte Carlo simulations were performed to explore dosing regimens that would maintain an inhibition of withdrawal responses or suppressed reinforcement under the under the influence of swallowing frequency. All simulated doses were NOS-adjusted. 1,000 virtual patients were created for each dose regimen to simulate the individual PK profiles. These simulated PK profiles were then used to determine %T > MSC for each virtual patient on the eighth day (at steady state). The proportion of patients who achieved different %T > MSC targets under each dose regimen was summarized as the probability of target attainment (PTA).

#### 3.5.2 Simulation of μ-opioid receptor occupancy

Model-based simulation was used to explore receptor occupancy at the steady state under different dose regimens. The simulation for PK profiles was identical as that for %T > MSC simulation (as above). The conversion from BUP plasma concentration to μORO followed an Emax model Eq. 8 (Nasser et al., 2014).

$$\mu ORO=\frac{E_{\max}∙C_{p}}{{EC}_{50}+C_{P}}$$ (8)

Where $E_{\max}$ is the maximum receptor occupancy (91.4%), ${EC}_{50}$ is the concentration of plasma BUP that leads to half of the maximum receptor occupancy (0.67 ng/mL ), and the target μORO is set to 70%.

#### 3.5.3 Simulation of naloxone

The plasma concentrations of NLX were simulated based on the established popPK model to explore the maximum concentration of NLX under all the investigated dosage regimens. According to the literature, the 2-h AUC of 550 ng/mL∙min after oral NLX, or an average plasma concentration of 4.6 ng/mL , was the threshold for reversing the effect of opioid receptor agonists and triggering withdrawal symptoms (Culpepper-Morgan et al., 1992). The dose regimen was considered a valid option only when the plasma concentration of NLX did not exceed this threshold at all times.

## 4 Results

### 4.1 Patient characteristics

A total of 46 Han Chinese males were included in this study. In addition, eight subjects were excluded because their vital signs or biochemical tests were outside the normal range and were clinically significant. The subjects in the single-dose group were all healthy volunteers, 12 individuals in each of the 4 mg and 8 mg groups, and 10 individuals in the 16 mg group. The 12 subjects in the multiple-dosing group were OUD individuals. In total, 34 healthy volunteers and 12 OUD subjects with 1874 plasma concentrations were collected, of which 699 were BUP samples, 691 were norBUP samples and 484 were NLX samples. The percentage of BQL within all subgroups of BUP and norBUP was less than 10% and treated with the M1 method. For NLX concentrations, 20.3% of single doses and 56.2% of repeat doses were BQL and were analyzed using the M3 method. There was no significant difference between healthy volunteers and OUD subjects in height, BMI and weight, except for the median age (26 years vs. 34 years). The observed number of swallows after dose administration ranged from 0 to 12, with high doses often accompanied by multiple swallows. All demographic characteristics of the included subjects were listed in Table 1.

**TABLE 1:** The demographic information of the study population.

Variables Median [min, Max]	4 mg	8 mg		16 mg	Overall
	Single dose	Multiple dose	Single dose	Single dose	
	(N = 12)	(N = 12)	(N = 12)	(N = 10)	(N = 46)
**Disease state**					
OUD patients	0	12	0	0	12
healthy volunteers	12	0	12	10	34
Age (years)	23.5 [21.0, 30.0]	34.0 [27.0, 44.0]	28.0 [20.0, 30.0]	26.0 [22.0, 30.0]	27.5 [20.0, 44.0]
Height (m)	1.73 [1.67, 1.88]	1.69 [1.64, 1.82]	1.73 [1.67, 1.83]	1.72 [1.60, 1.74]	1.72 [1.60, 1.88]
BMI (kg/m^2^)	22.6 [19.0, 24.9]	21.5 [18.7, 24.2]	22.5 [19.6, 24.6]	20.8 [20.2, 23.9]	22.1 [18.7, 24.9]
Weight (kg)	69.0 [54.0, 88.0]	64.0 [53.0, 70.2]	66.0 [60.0, 78.0]	60.5 [53.0, 70.0]	65.0 [53.0, 88.0]
White blood cell (10^9^/L)	5.45 [3.40, 7.40]	7.35 [4.79, 11.0]	5.95 [4.00, 6.60]	5.20 [4.80, 7.10]	5.90 [3.40, 11.0]
Red blood cell (10^13^/L)	4.97 [4.42, 5.33]	4.89 [4.51, 5.92]	4.75 [3.70, 5.28]	4.54 [3.81, 5.18]	4.78 [3.70, 5.92]
Hemoglobin (g/L)	146 [129, 165]	156 [137, 168]	145 [111, 158]	139 [127, 175]	146 [111, 175]
Platelet (10^9^/L)	226 [149, 257]	266 [152, 443]	202 [165, 342]	268 [195, 419]	227 [149, 443]
ALT (U/L)	11.5 [4.30, 28.4]	43.9 [20.6, 157]	13.3 [6.80, 124]	9.10 [7.20, 33.8]	14.8 [4.30, 157]
AST (U/L)	14.1 [8.40, 19.8]	31.8 [17.4, 86.0]	12.0 [1.90, 41.4]	16.6 [8.50, 20.2]	17.2 [1.90, 86.0]
Total bilirubin (μmoL/L)	16.9 [12.5, 27.2]	17.7 [6.00, 24.2]	10.7 [6.70, 18.1]	14.1 [6.30, 32.2]	13.3 [6.00, 32.2]
CrCL (mL/min)	131 [102, 198]	123 [106, 144]	133 [103, 189]	115 [96.9, 165]	124 [96.9, 198]
Number of swallowing	3.50 [0, 8.0]	—	2.0 [0, 3.0]	6.5 [2.0, 12.0]	2.0 [0,12.0]

∗ALT, alanine aminotransferase; AST, aspartate aminotransferase; CrCL, creatinine clearance calculated with Cockcroft-Gault formula.

### 4.2 Non-compartmental pharmacokinetic analysis

The estimated PK parameters of each dose group were summarized in Supplementary Table S1. In the single-dose group of BUP and norBUP, the 16 mg group of BUP had a smaller dose normalization AUC (4 mg:8 mg:16 mg median = 3869:3928:2656 (ng*h/L)/mg) and dose normalization $C_{\max}$ [4 mg:8 mg:16 mg median = 563:583:368 (ng/L)/mg], while the opposite results were found in norBUP. By comparing ${AUC}_{168-192h}$ of multiple-dosing with ${AUC}_{0-24h}$ of the 8 mg single-dose group, it was found that neither BUP nor norBUP did accumulate *in vivo* after repeat administrations. For NLX, the dose-normalized AUC and the dose-normalized $C_{\max}$ decreased as dose increased. NLX did not accumulated in the body after repeated dosing. Ethnic sensitivity analysis showed that both Chinese and Puerto Ricans had a similar $T_{\max}$ (1.52 h vs. 1.45 h). However, the dose-normalized $C_{\max}$ and AUC of BUP in Chinese were only about half of that in Puerto Ricans, which was consistent with higher observed clearance in Chinese (166 L/h vs. 270 L/h ) (Table 2).

**TABLE 2:** The non-compartmental analysis results of Chinese and Puerto Rico.

	Chinese	Puerto rico
$T_{\max}$ (h)	1.52 ± .81	1.45 ± 0.69
$C_{\max}$ /Dose ( $ng∙L^{-1}∙{mg}^{-1}$ )	583 ± 248	1,060 ± 790
AUC/Dose ( $ng∙h∙L^{-1}∙{mg}^{-1}$ )	1897 ± 779	3840 ± 2350

### 4.3 Buprenorphine parent-metabolite model

**FIGURE 1:**
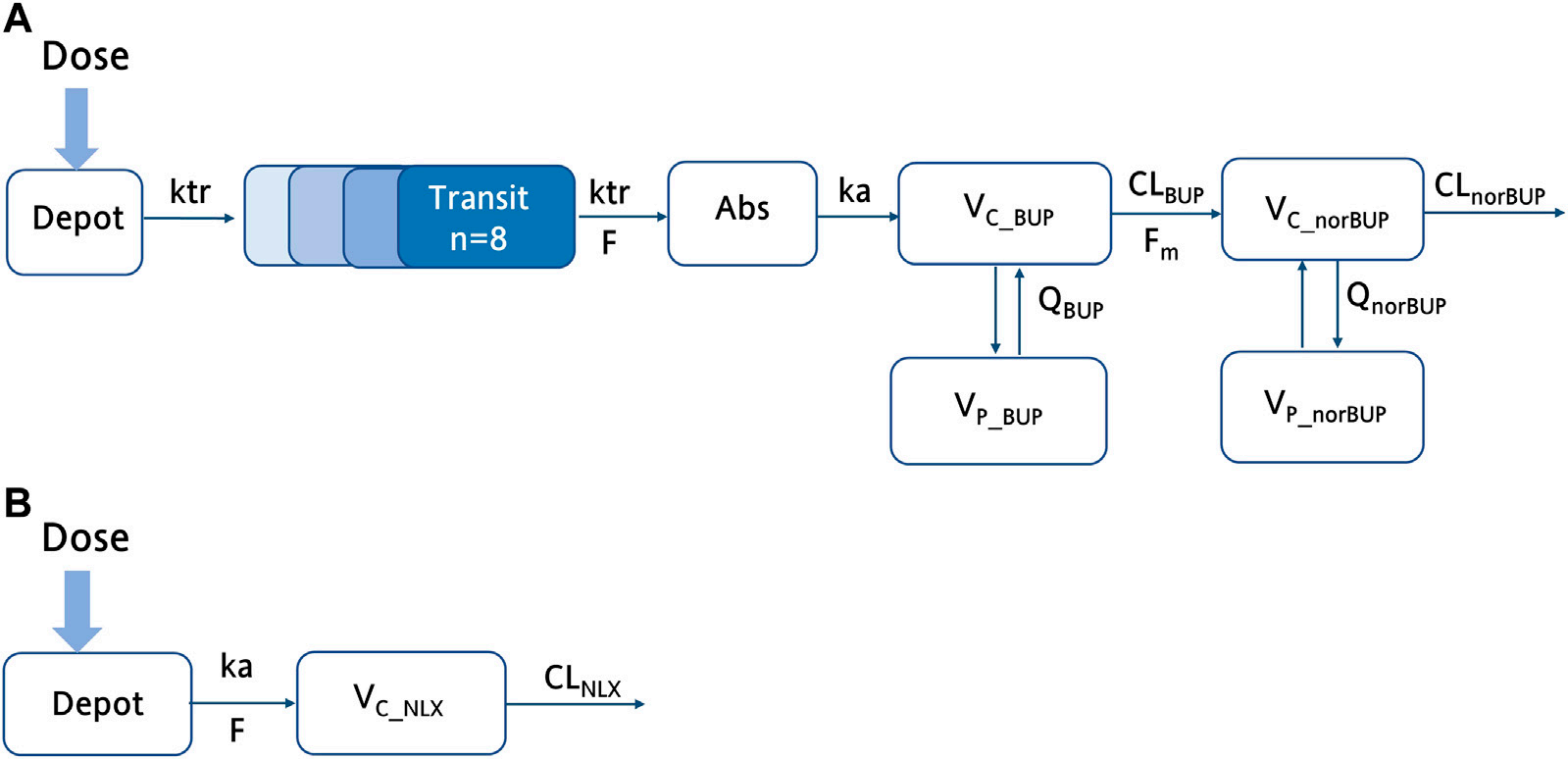
The schematic structure of the final buprenorphine-norbuprenorphine and naloxone model. **(A)** The schematic diagram of the buprenorphine parent-metabolite model, including wo compartment model for buprenorphine, two compartment model for norbuprenorphine, and transit compartments for absorption; **(B)** The schematic diagram of the NLX with one compartment model. *ka*, the absorption rate constant; *F*, bioavailability; $V_{C\_BUP}{/V}_{C\_norBUP}{/V}_{C\_NLX}$ , the central volume of distribution; $V_{P\_BUP}{/V}_{P\_norBUP}$ , the peripheral volume of distribution; $Q_{P}/Q_{m}$ , the intercompartmental clearances; ${CL}_{BUP}/{CL}_{norBUP}/{CL}_{NLX}$ , clearance; *ktr*, the transit rate constant of BUP; $F_{m}$ , the fraction of the BUP transformed to norBUP.

For buprenorphine, the two-compartment model could describe the data better than the one-compartment model (∆AIC = −588.7). In addition, compared with the time lag model, the transit absorption model resulted in a further decrease in AIC (∆AIC = −74.8). A two-compartment model with transit compartments for absorption was finally selected as the structure model to describe the PK profile of BUP (see Figure 1A). As an important covariate of interest and mechanism, the NOS was preferentially considered for the structure model. The inclusion of NOS in bioavailability significantly improved the model, with 16.92 points reduction in OFV (*p* < 0.001). In the observed range of 0–12 swallows, bioavailability decreased by 3.50% for each additional swallow. For norBUP, a two-compartment model could describe the data better than a one-compartment model (∆AIC = −765.1). Due to the lack of intravenous administration data, the fraction of the BUP transformed to norBUP ( $F_{m}$ ) was not identifiable and thus was fixed to one to reflect the fact that metabolite-related PK parameters were apparent. IIV was estimated for all parameters by the exponential model except for the absorption rate constant (*k*_*a*_). For the residual variability, proportional errors were estimated for BUP and norBUP separately. The key model development steps were summarized in Supplementary Table S3.

The covariates investigated included disease state (healthy volunteers or OUD subjects), age, weight, height, and dosage administered. Since weight was highly correlated with height, only the disease state, age, weight, and dosage were selected for the SCM procedure. After the forward selection and backward elimination, the dosage (4 mg, 8 mg, and 16 mg) on $F_{m}$ was confirmed as another significant covariate (∆OFV = −34.0, *p* < .001). Compared to 4 mg and 8 mg, participants who received 16 mg drugs had higher $F_{m}$ (1.98 vs. 1), suggesting that other elimination pathways of BUP may be saturated at high doses. However, there were no differences in other parameters between healthy volunteers and OUD subjects.

**FIGURE 2:**
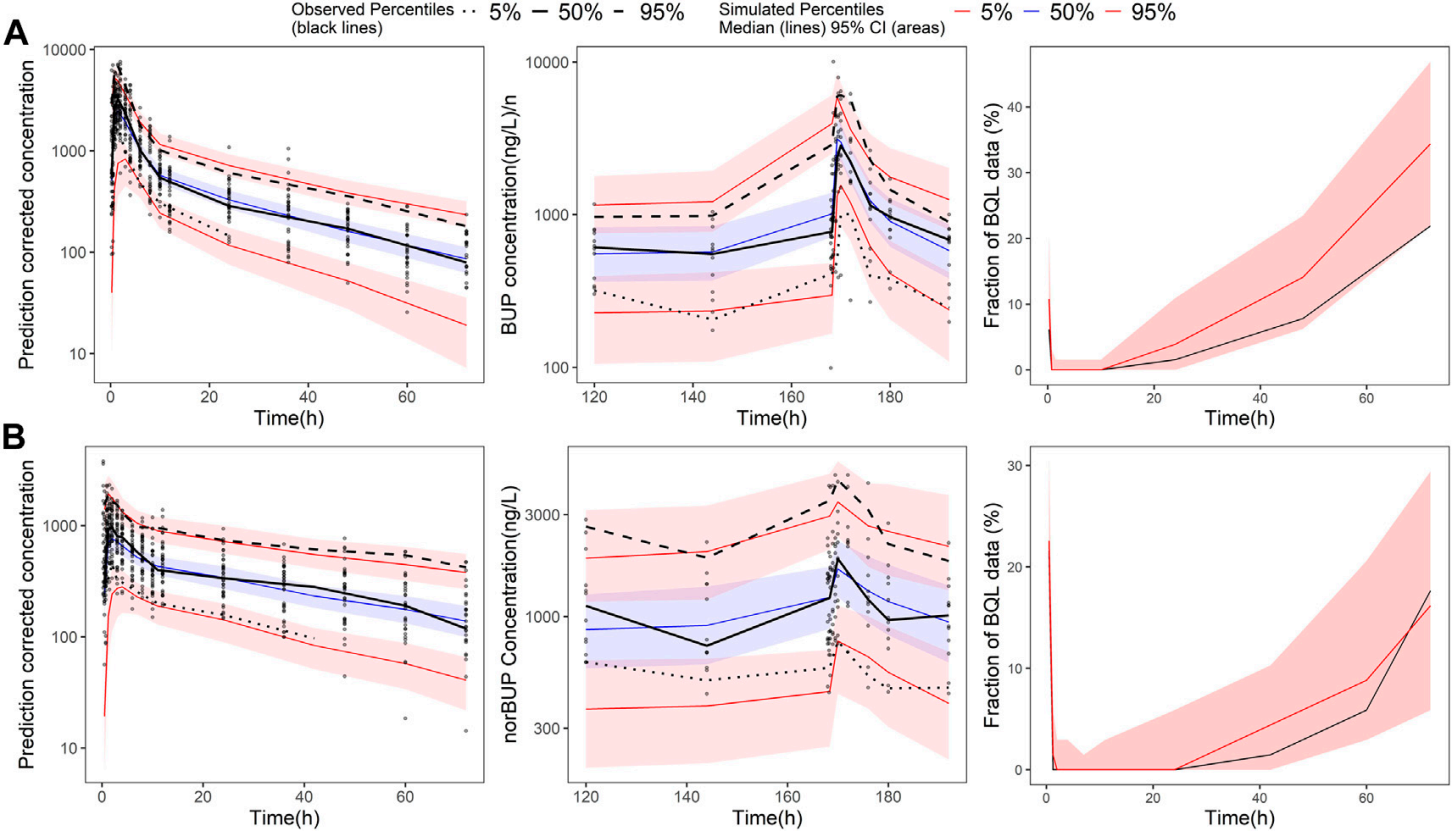
Prediction-corrected visual predictive check of the final buprenorphine-norbuprenorphine population pharmacokinetic model. The first row shows the results for buprenorphine (BUP), and the second row shows the results for norbuprenorphine (norBUP). **(A)** pcVPC of BUP for single dose study; **(B)** VPC of BUP for multiple dose study; **(C)** the fraction of BQL data over time in BUP; **(D)** pcVPC of norBUP after a single dose; **(E)** VPC of norBUP after multiple doses; **(F)** the fraction of BQL data over time in norBUP. Circles represent observed data. Black lines represent the 5% (dashed), 50% (solid), and 95% (dashed) percentiles of the observed data. Shaded areas represent 95% confidence intervals of the median 5% (red), 50% (blue), and 95% (red) percentiles of the predicted concentrations. For C and G, the red and black lines represent the median of predicted and observed BQL fraction, respectively. Shaded areas represent 90% prediction intervals.

All the parameters of the final model were estimated with acceptable precision (Table 2). The GOF result of the final parent-metabolite model (Supplementary Figures S5, S6) demonstrated a good fit of the final model. The success rate of bootstrap was 90.1%, and the estimated values of the final model parameters were close to the median and within the 95% CI from the non-parametric bootstrap (see Table 2). The pcVPC result (Figure 2) suggested that most of the observed values were contained within the 90% prediction intervals, indicating that the final model has sufficient predictive ability. The simulated median of the BQL fraction was consistent with the observed BQL fraction over time, suggesting that the M1 method was sufficient in handling BQL data for the buprenorphine parent-metabolite model. The result of the median and 95% CI of NPC also agreed with the observed data (Supplementary Table S2).

### 4.4 Naloxone model

Compared with the two-compartment model, a one-compartment model was sufficient to describe the observed concentrations of NLX (∆AIC = −8.06). According to previous study, the bioavailability of NLX sublingual tablet was fixed to 0.01 (McDonald et al., 2018). The IIV term was estimated for ${CL}_{NLX}$ and $V_{C\_NLX}$ by the exponential model. The residual error was best described by a combined proportional and additive error model. The model structure was shown in Figure 1B and the model development process was shown in Supplementary Table S3.

**FIGURE 3:**
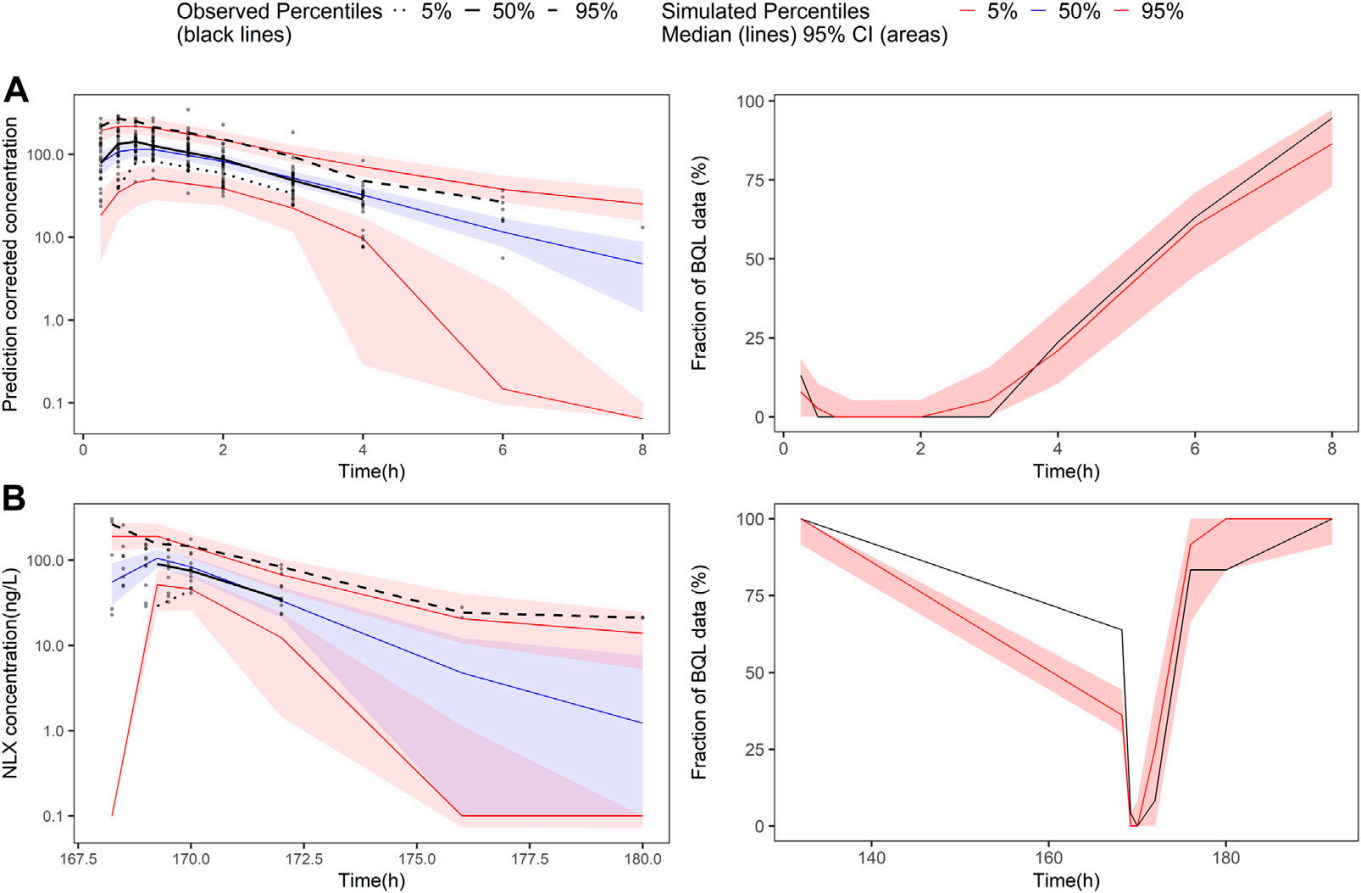
Prediction-corrected visual predictive check and visual predictive check of the final naloxone population pharmacokinetic model. The first row shows the results for single dose study, and the second row shows the repeat dose study. **(A)** pcVPC results for single dose study; **(B)** the fraction of BQL data over time after a single dose; **(C)** VPC results for multiple dose study; **(D)** the fraction of BQL data over time after multiple doses. Circles represent observed data. Black lines represent the 5% (dashed), 50% (solid), and 95% (dashed) percentiles of the observed data. Shaded areas represent 95% confidence intervals of the median 5% (red), 50% (blue), and 95% (red) percentiles of the predicted concentrations. For B and D, the red and black lines represent the median of predicted and observed BQL fraction, respectively. Shaded areas represent 90% prediction intervals.

In the covariate analysis, age, weight, NOS, dosage, and disease state (healthy volunteers or OUD subjects) were tested. After SCM, dosage on bioavailability was a significant covariate, resulting in a decrease in OFV by 12.02 points (*p* < 0.001). The final model parameters were estimated with acceptable precision (Table 3). The GOF plots for the final model of NLX showed good agreement between model prediction and observed data. No obvious trend was observed in the NPDE analysis (Supplementary Figure S8). The 95% CI of bootstrap included the parameter estimates of the final model, and the median was close to the final parameter estimates, with a success rate of 96.8% (Table 4). Furthermore, VPC result indicated the final model well described the general trend of the observed data and adequately captured the variability in this study (Figure 3). According to the NPC results, the median and 95% CI in the model-based predictions agreed with the observed data (Supplementary Table S2).

**TABLE 3:** Parameter estimates of the final naloxone population pharmacokinetic model and bootstrap results.

Parameter	Final estimation (%RSE) [Shrinkage]	Bootstrap median [95% CI]
${CL}_{NLX}$ , L/h	205 (5%)	205.7 [185.5–225.9]
$V_{C\_NLX}$ , L	104 (17%)	104.6 [76.0–144.6]
ka,/h	0.585 (4%)	0.586 [0.540–0.640]
F	0.01 FIX (McDonald et al., 2018)	—
Exponent for dose on F	−0.36 (29%)	−0.36 [−0.58–0.16]
Residual error		
σ_prop_ (%CV)	20.8% (16%) [16%]	20.5% [13.0%–26.7%]
σ_additive_ (sd)	9.24 (13%) [16%]	9.10 [6.45–14.10]
IIV (%CV)		
${CL}_{NLX}$	31.2% (14%) [6%]	30.30% [21.60%–39.10%]
$V_{C\_NLX}$	102.5% (12%) [6%]	101.44% [59.60%–159.80%]

∗%RSE, percent relative standard error; CI, confidence interval; % CV, coefficient of variation; SD, standard error; σ_prop,_ proportional error; σ_additive_, additional error.

**TABLE 4:** Parameter estimates of the final buprenorphine-norbuprenorphine population pharmacokinetic model and bootstrap results.

Parameter	Final estimation (%RSE)] [Shrinkage]	Bootstrap median [95% CI]
${CL}_{BUP}$ /F, L/h	270 (10%)	270.4 [244.9–297.7]
$V_{C\_BUP}$ /F, L	377 (35%)	377.2 [262.1–566.1]
$V_{P\_BUP}$ /F, L	5879 (13%)	5876.2 [5003.4–7029.0]
ka,/h	0.397 (6%)	0.40 [0.35–0.45]
$Q_{BUP}$ /F, L/h	404 (14%)	406 [341.0–487.2]
MTT, h	0.234 (8%)	0.23 [0.199–0.268]
The effect of NOS on F	−0.035 (27%)	−0.036 [−0.058–0.01]
${CL}_{norBUP}$ /Fm, L/h	22.2 (68%)	22.5 [4.68–48.2]
$V_{C\_norBUP}$ /Fm, L	264 (15%)	265 [223–314]
$V_{P\_norBUP}$ /Fm, L	5170 (13%)	5142 [4186–6283]
$Q_{norBUP}$ /Fm, L/h	705 (21%)	705 [570–870]
Fm increment of 16 mg	1.98 (15%)	1.97 [1.49–2.69]
Residual error (%CV)		
σ_prop_ (BUP)	28.1% (3%) [10%]	27.9% [24.7%–31.1%]
σ_prop_ (norBUP)	22% (3%) [10%]	21.7% [19.2%–24.5%]
IIV (%CV)		
${CL}_{BUP}$ /F	33.2% (20%) [3%]	32.5% [26.5%–39.2%]
$V_{C\_BUP}$ /F	135.6% (8%) [6%]	130.5% [74.0%–193.1%]
$V_{P\_BUP}$ /F	41.4% (23%) [18%]	39.8% [27.1%–52.0%]
$Q_{BUP}$ /F	49.4% (16%) [10%]	48.6% [34.3%–62.2%]
MTT	37.5% (22%) [19%]	37.3% [20.6%–65.1%]
${CL}_{norBUP}$ /Fm	119.6% (47%) [44%]	113.0% [2.1%–217.3%]
$V_{C\_norBUP}$ /Fm	50.5% (18%) [1%]	48.9% [39.2%–58.5%]
$V_{P\_norBUP}$ /Fm	45.1% (27%) [23%]	43.5% [30.1%–56.1%]
$Q_{norBUP}$ /Fm	56.7% (23%) [11%]	55.0% [36.4%–72.0%]

∗%RSE, percent relative standard error; CI, confidence interval; %CV, coefficient of variation, MTT, the mean transit time of BUP traveling from the first transit compartment to the absorption compartment.

### 4.5 Dose optimization *via* Monte Carlo simulations

**FIGURE 4:**
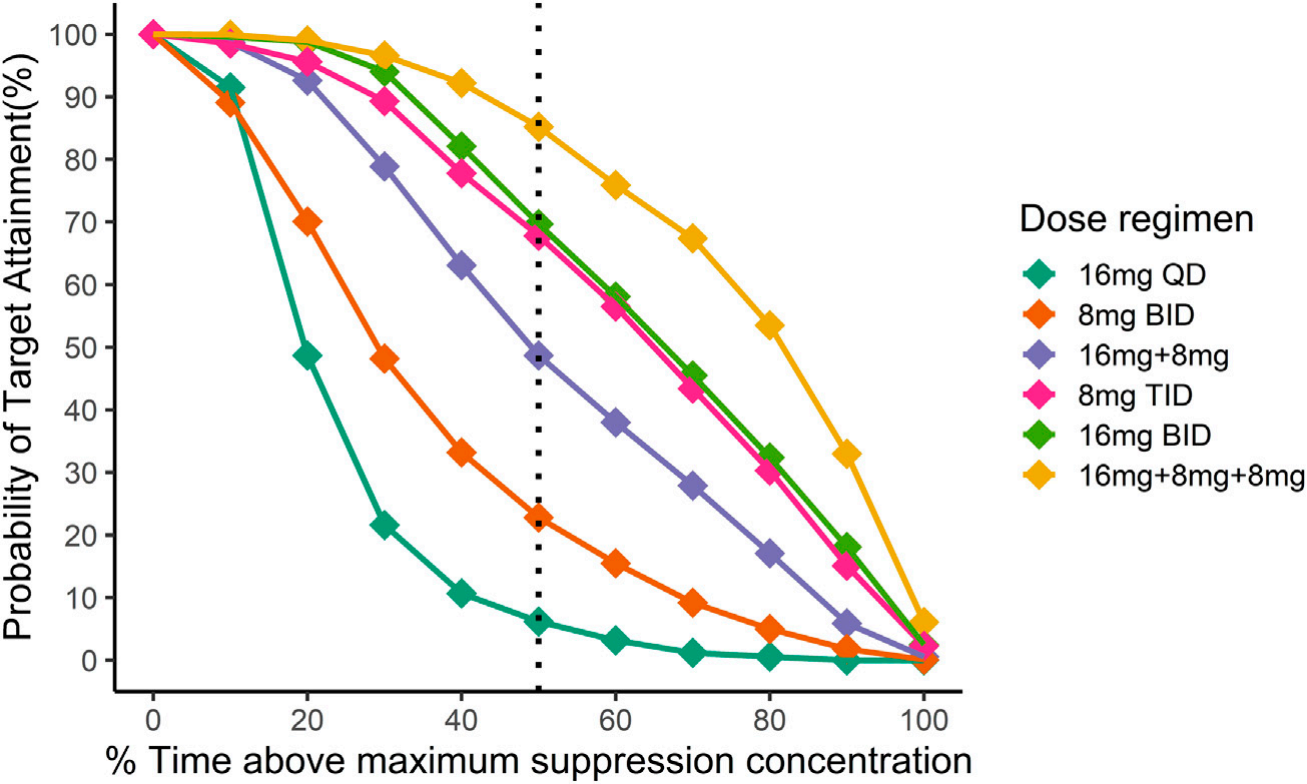
Simulation of the time above minimum suppression concentration in Chinese after different dosing regimens. Different colors indicate different dosing regimens. The *x*-axis is the mean proportion of the time when the plasma concentration exceeds the target concentration at the steady state, and the *y*-axis is the proportion of the population that reaches the %T > MSC target. Then black dotted line indicates the fraction of time above maximum suppression concentration equal to 50%.

Figure 4 illustrated the probability of target attainment (PTA) for different %T > MSC targets under various dose regimens at steady state. With the target of %T > MSC equal to 50%, the PTA of 16 mg BID, 8 mg TID and 16 mg + 8 mg + 8 mg daily dose regimens all approached 70%.

**FIGURE 5:**
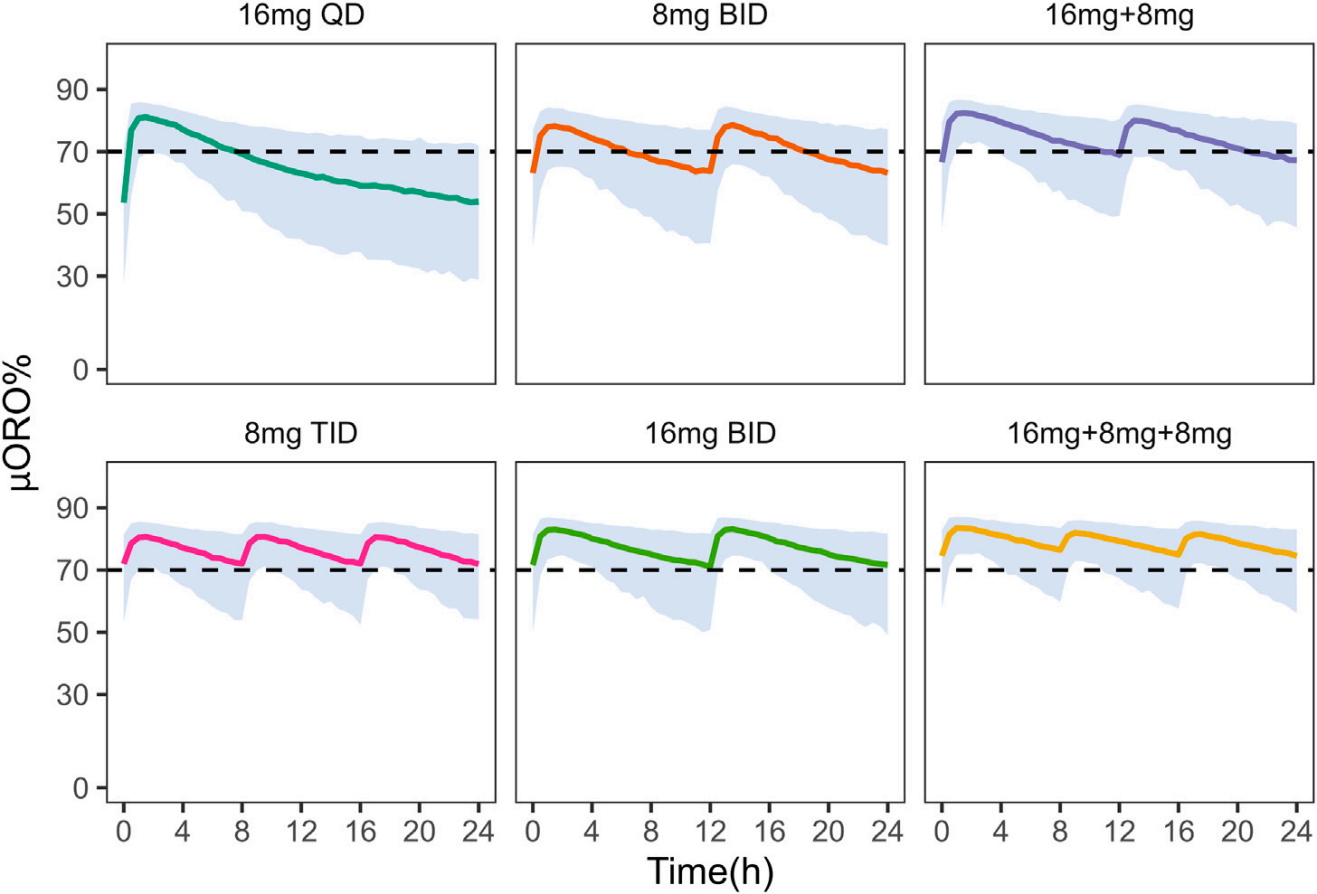
Simulation of μ-opioid receptor occupancy in Chinese after different dosing regimens. The shaded areas represent the 90% prediction intervals of the simulated data. Above the black dashed line, the receptor occupancy exceeds 70%.

In addition, these dosing regimens showed comparable results in μ-opioid receptor occupancy ( μORO ) at the steady state (Figure 5) and all regimens achieved and maintained the desired μORO ( ≥ 70%). Compared to the other two dose regimens with higher daily doses (24 mg vs. 32 mg), 8 mg TID was sufficient to achieve maximum efficacy.

**FIGURE 6:**
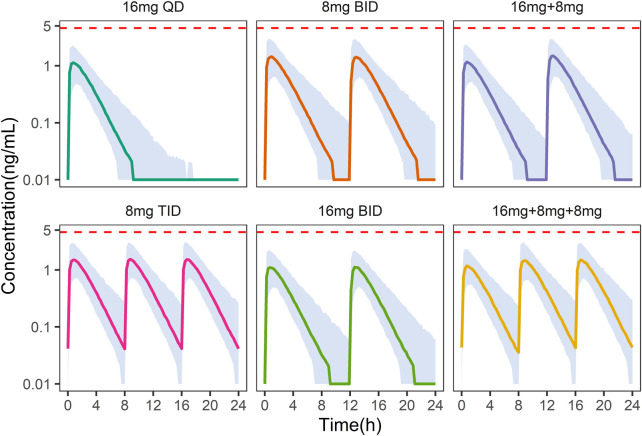
Simulation of plasma naloxone concentration in Chinese after different dosing regimens. Different colors indicate different dosing regimens. The shaded areas represent the 90% prediction intervals of the simulated data. The dashed red line indicates a concentration of 4.6 ng/mL, above which withdrawal may occur.

On the other hand, under the 8 mg TID dosing regimen, the peak concentration of NLX at the steady state was below the withdrawal reaction threshold (4.6 ng/mL ) in more than 95% of patients (Figure 6). Hence, 8 mg TID was recommended as a safe and efficacious dose regimen for Chinese OUD patients.

## 5 Discussion

This study was based on data from healthy Chinese volunteers and OUD subjects after single and repeated administration of BUP/NLX sublingual tablets. In this study, we developed two popPK models to clarify the pharmacokinetic profile of BUP/NLX sublingual tablets. The PK characteristics of BUP-norBUP was described using a four-compartment model with transit-compartments for the absorption processes. A one-compartment model was selected to describe NLX data. No differences were found between healthy volunteers and OUD subjects in the pharmacokinetics of BUP, norBUP and NLX. Model-based simulations suggest that 8 mg TID was the optimised dosage for combined efficacy and safety in Chinese OUD subjects.

According to the NCA results, the $C_{\max}$ of BUP had low dose-proportionality, indicating that the bioavailability decreased as doses increased. This phenomenon can be explained by the positive correlation of NOS with increasing dosage, suggesting the drug had entered the gastrointestinal tract and underwent first-pass elimination. Each additional swallow resulted in a 3.5% reduction in BUP bioavailability over the observed frequency. Contrary to our initial hypothesis, $F_{m}$ was 2.17-fold higher in the high-dose group (16 mg) than in the low- and medium-dose groups (4 mg and 8 mg). A possible explanation for this phenomenon is that other metabolic pathways (e.g., metabolism to buprenorphine glucuronide) of BUP was saturated at high doses, thus facilitating the metabolism of BUP to norBUP. Unlike BUP, dosage rather than NOS was an important covariate of NLX bioavailability, with higher doses resulting in lower bioavailability. Higher doses may reduce the solubility of the tablets in saliva, resulting in a decrease in bioavailability (Chiang and Hawks, 2003). It may be explained by the low bioavailability of NLX sublingual itself, which was not significantly affected by swallowing.

This was the first popPK study to describe the PK characteristics of BUP and norBUP in a Chinese population. The apparent *CL* of BUP was consistent with a previous NCA study of sublingual BUP/NLX in Chinese healthy subjects (8 mg: 270 L/h vs. 275 L/h ) (Dong et al., 2019). The AUC_0-72_ of BUP in each dose groups were slightly greater than the results of Ciraulo et al. 2006 (8 mg: 3,928 $ng∙h∙L^{-1}∙{mg}^{-1}$ vs. 2,903 $ng∙h∙L^{-1}∙{mg}^{-1})$ , while the results of NLX were similar to those of Western subjects (8 mg: 39 $ng∙h∙L^{-1}∙{mg}^{-1}$ vs. 48 $ng∙h∙L^{-1}∙{mg}^{-1}$ ). In contrast, multiple-dose-related BUP accumulation was not observed in our data. The model established in Puerto Rico considered both the metabolism of BUP as buprenorphine-3-glucuronide and norBUP, whereas only the metabolite norBUP was considered in our model. Comparing the rate of BUP metabolism in our model ${CL}_{BUP}$ and the rate of BUP metabolism to two products in the Puerto model, the result was that the rate of BUP metabolism in the Chinese population was greater than that of Puerto Ricans (270 vs. 166 L/h ). The larger clearance rate was consistent with the NCA results, in which the Chinese had half the dose-normalized Cmax and dose-normalized ${AUC}_{0-8h}$ of the Puerto Ricans ( Cmax :583 $ng∙L^{-1}∙{mg}^{-1}$ vs. 1,060 $ng∙L^{-1}∙{mg}^{-1}$ ; ${AUC}_{0-8h}$ : 1,897 $ng∙h∙L^{-1}∙{mg}^{-1}$ vs. 3,840 $ng∙h∙L^{-1}∙{mg}^{-1}$ ). Additional research is needed to clarify the physiological mechanisms involved.

In China and other countries, such as Iran and United States, daily doses of ≤ 24 mg appeared to be the widely accepted treatment protocol for OUD subjects (Ciraulo et al., 2006; Ziaaddini et al., 2018; Dong et al., 2019). The trough levels of BUP obtained in the multiple-dose group (8 mg QD) in our study were less than 3 ng/mL , indicating that most recruited OUDs may experience withdrawal symptoms before taking the daily maintenance dose, thus requiring higher doses to maintain adequate plasma concentration. To explore the level of plasma concentration after multiple doses reached a steady state, we chose a series of higher doses for simulation based on the final model. The %T > MSC was used to represent the proportion of time in which the plasma concentration of 3 ng/mL BUP can be maintained on any day after homeostasis has been achieved at different dosages. With a target of %T > MSC equal to 50%, PTA approached 70% for each of the 16 mg BID, 8 mg TID, and 16 mg + 8 mg + 8 mg daily dose regimens. Compared with %T > MSC; μORO may be a more intuitive indicator for evaluating drug efficacy. After simulation of μORO with the same dosage, 8 mg TID, 16 mg BID and daily doses of 16 mg + 8 mg + 8 mg all achieved and maintained μORO above 70%. A comprehensive evaluation of safety (the concentration of NLX <4.6 ng/mL ) and efficacy, 8 mg TID was a more suitable initial dose for Chinese OUD subjects and was consistent with the widely accepted dosing protocol. The dosage can be adjusted in the follow-up treatment according to the patient’s sensitivity and efficacy. In addition, considering the adverse reactions brought about by higher doses, a possible method is to adjust the administration time. Keeping μORO below 70% of the time occurring during sleep may feasible to prevent OUD subjects from experiencing discomfort or even relapse due to withdrawal.

There are some limitations to this study. Due to the lack of actual bioavailability of BUP and the metabolism ratio of BUP to norBUP, only apparent parameters could be estimated. The measurement of BUP and norBUP in urine may be added in subsequent trials to compensate for this deficiency. In addition, the missing of female pharmacokinetic data in clinical trial limits the capability of the developed model in describing human pharmacokinetics in a more general sense. Hence, further studies may be warranted to investigate the impact of sex on human pharmacokinetics.

## 6 Conclusion

In conclusion, a popPK model for BUP/NLX sublingual tablets was developed to describe the time course of the three compounds (BUP, norBUP and NLX) in Chinese healthy volunteers and individuals with opioid use disorders. The model-based dose optimization recommended 8 mg TID as the initial dose regimen for Chinese OUD patients with satisfactory efficacy and safety profiles.

## Data Availability

The raw data supporting the conclusion of this article will be made available by the authors, without undue reservation.
