# Supplementary material for: Population pharmacokinetics of buprenorphine and naloxone sublingual combination in Chinese healthy volunteers and patients with opioid use disorder: Model-based dose optimization
Source: Front Pharmacol. 2023 Jan 19;14:1089862. doi: 10.3389/fphar.2023.1089862 (PMC9893638; doi:10.3389/fphar.2023.1089862)
Supplement: Supplementary file 1 [file Table1.DOCX]

Supplementary Material

**Table S1.** The non-compartmental analysis results.

|  | **single dose**  **(0-72h)**  mean±SD | | | **multiple dose**  **(168-192h)**  mean±SD |
| --- | --- | --- | --- | --- |
| **BUP** | **4mg** | **8mg** | **16mg** | **8mg** |
| $\mathbf{T}_{\mathbf{max}}$ | 1.48±0.46 | 1.52±0.85 | 1.22±0.60 | 1.83±1.93 |
| $\mathbf{C}_{\mathbf{max}}$**/Dose** | 563±244 | 583±259 | 368±164 | 501±324 |
| **AUC/Dose** | 3869±1095 | 3928±1818 | 2656±568 | 4004±1780 |
| **norBUP** | **4mg** | **8mg** | **16mg** | **8mg** |
| $\mathbf{T}_{\mathbf{max}}$ | 2.02±1.01 | 2.06±3.18 | 0.79±0.41 | 3.33±1.23 |
| $\mathbf{C}_{\mathbf{max}}$**/Dose** | 153±66 | 150±73 | 237±91 | 286±146 |
| **AUC/Dose** | 3498±1621 | 3191±1392 | 4591±1630 | 4158±2226 |
| **NLX** | **4mg** | **8mg** | **16mg** | **8mg** |
| $\mathbf{T}_{\mathbf{max}}$ | 0.85±0.27 | 1.25±0.94 | 0.58±0.21 | 1.13±0.67 |
| $\mathbf{C}_{\mathbf{max}}$**/Dose** | 27±9 | 20±9 | 17±7 | 18±10 |
| **AUC/Dose** | 59±31 | 39±16 | 40±16 | 45±28 |

*****$T_{\max}$, h; $C_{\max}$/Dose, $ng\cdot L^{-1}\cdot\mathrm{mg}^{-1}$; AUC/Dose, $ng\cdot h\cdot L^{-1}\cdot\mathrm{mg}^{-1}$

**Table S2.** The numerical predictive check results.

|  | Observe mean | | NPC result median[95%CI] | |
| --- | --- | --- | --- | --- |
| BUP | $\mathbf{AUC}_{\mathbf{0-72h}}$ | $\mathbf{C}_{\mathbf{max}}$ | $\mathbf{AUC}_{\mathbf{0-72h}}$ | $\mathbf{C}_{\mathbf{max}}$ |
| 4mg | 15474 | 2253 | 13103 [10832-15453] | 1643[1259-2115] |
| 8mg | 31426 | 4667 | 2768[23264-32262] | 3510[2709-4437] |
| 16mg | 42495 | 5893 | 45388[37633-54373] | 5795[4392-7462] |
| 8mg(multiple-dose) | 32032(0-24h) | 4011 | 31222[25910-37668] | 4108[3285-5099] |
| norBUP | $\mathbf{AUC}_{\mathbf{0-72h}}$ | $\mathbf{C}_{\mathbf{max}}$ | $\mathbf{AUC}_{\mathbf{0-72h}}$ | $\mathbf{C}_{\mathbf{max}}$ |
| 4mg | 13993 | 612 | 10949[8513-14227] | 479[360-657] |
| 8mg | 25529 | 1204 | 23826[18140-30043] | 1041[773-1397] |
| 16mg | 103429 | 3799 | 77032[57658-99500] | 3400[2488-4675] |
| 8mg(multiple-dose) | 33261(0-24h) | 2291 | 30246[23163-41118] | 2187[1692-2860] |
| NLX | $\mathbf{AUC}_{\mathbf{0-72h}}$ | $\mathbf{C}_{\mathbf{max}}$ | $\mathbf{AUC}_{\mathbf{0-72h}}$ | $\mathbf{C}_{\mathbf{max}}$ |
| 4mg | 235 | 108 | 244[201-297] | 97[77-119] |
| 8mg | 311 | 158 | 379[317-453] | 147[117-183] |
| 16mg | 642 | 277 | 590[490-719] | 228[177 -289] |
| 8mg(multiple-dose) | 359(0-24h) | 148 | 420[330-520] | 142[114-174] |

*AUC: $ng \cdot L^{-1}\cdot h$; $C_{\max}$: $ng/L$

**Table S3.** Brief summary of the buprenorphine-norbuprenorphine model development path.

| Model number | Reference model | | | AIC | ΔAIC | Description |
| --- | --- | --- | --- | --- | --- | --- |
| 1 (BUP start) | | - | 9480.6 | | - | one-compartment model |
| 2 | | 1 | 8891.9 | | -588.7 | two-compartment model |
| 3 | | 1 | 8900.0 | | -580.6 | three-compartment model |
| 4 | | 2 | 8783.6 | | -108.3 | two-compartment TLAG model |
| 5 | | 2 | 8712.8 | | -179.1 | two-compartment 8-transit model |
| 6 | | 4 | 8708.8 | | -74.8 | two-compartment 8-transit model,  IIV-ka fix to 0 |
| 6 | | 5 | 8706.8 | | -6 | two-compartment 8-transit model,  IIV-ka fix to 0, only prop. Error |
| 7 (BUP final) | | 6 | 8691.9 | | -14.9 | two-compartment 8-transit model,  IIV-ka fix to 0, only prop. error,  NOS on F |
| 8 (norBUP start) | | - | 17603.8 | | - | one-compartment model |
| 9 | | 8 | 16838.7 | | -765.1 | two-compartment model |
| 10 | | 8 | 16847.4 | | -756.4 | three-compartment model |
| 11 | | 9 | 16821.7 | | -17 | two-compartment model, IIV-$\mathrm{CL}_{\mathrm{norBUP}}$ fix to 0, only prop. error |
| 12 (norBUP final) | | 10 | 16800.3 | | -21.4 | two-compartment model, dose on Fm |
| 13 (BUP-norBUP final) | | 12 | 16700.6 | | -99.7 | buprenorphine-norbuprenorphine model, NOS on F, dose on Fm |
| 1(NLX first) | | - | | 2696.7 | - | one-compartment model |
| 2 | | 1 | | 2704.7 | 8 | two-compartment model |
| 3 | | 1 | | 2702.7 | 6 | three-compartment model |
| 4 | | 2 | | 2695.1 | -9.6 | two-compartment model, IIV-ka fix to 0 |
| 5(NLX final) | | 4 | | 2685.1 | -10 | two-compartment model,  IIV-ka fix to 0, dose on F |

*AIC, Akaike information criterion; NOS, number of swallows; F, bioavailability, Fm, the fraction of the BUP transformed to norBUP.


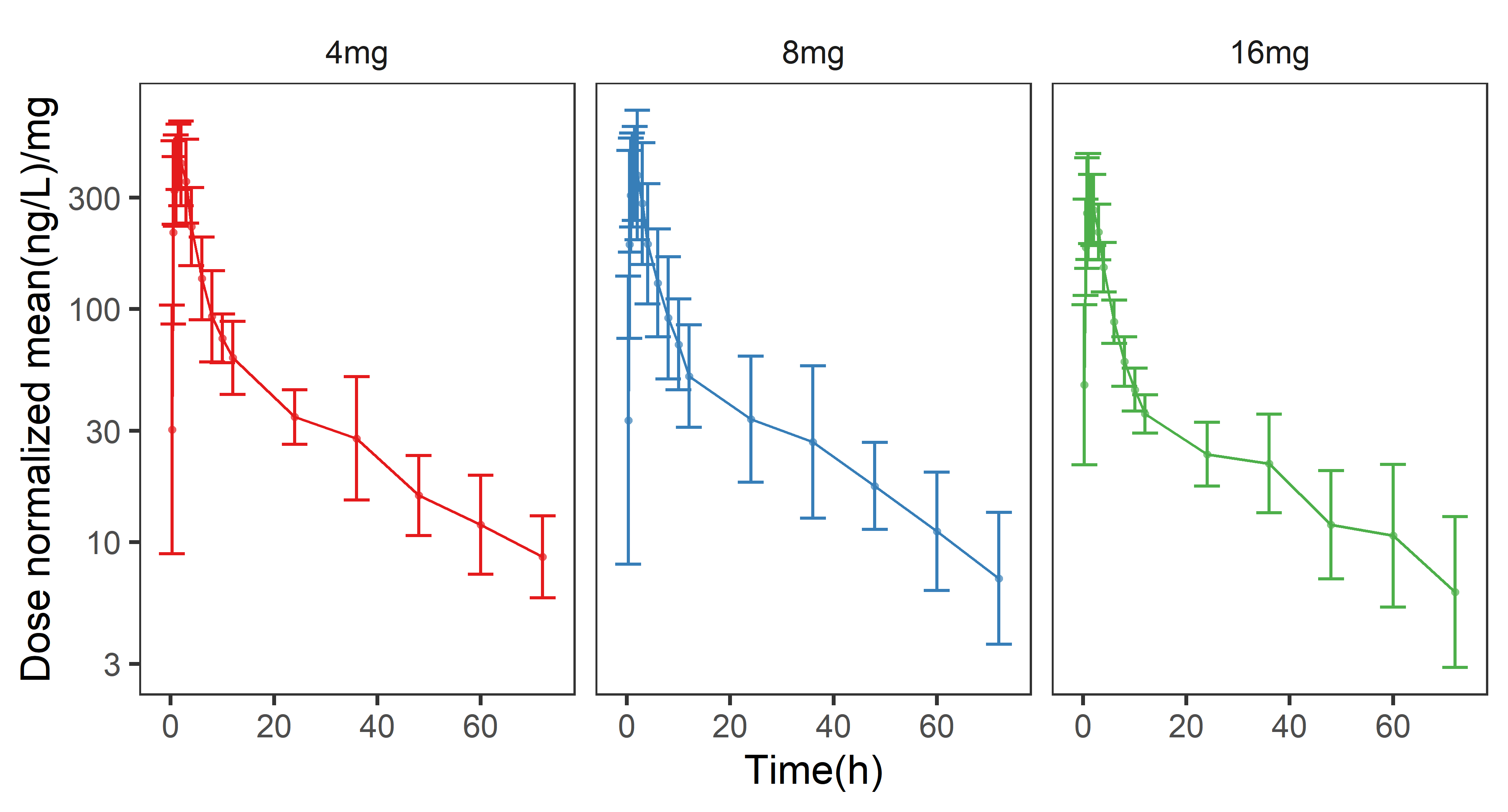


**Figure S1.** Dose normalized geometric mean of concentration-time graph for single dose of buprenorphine. The scale of y axis is log and of x is normal.


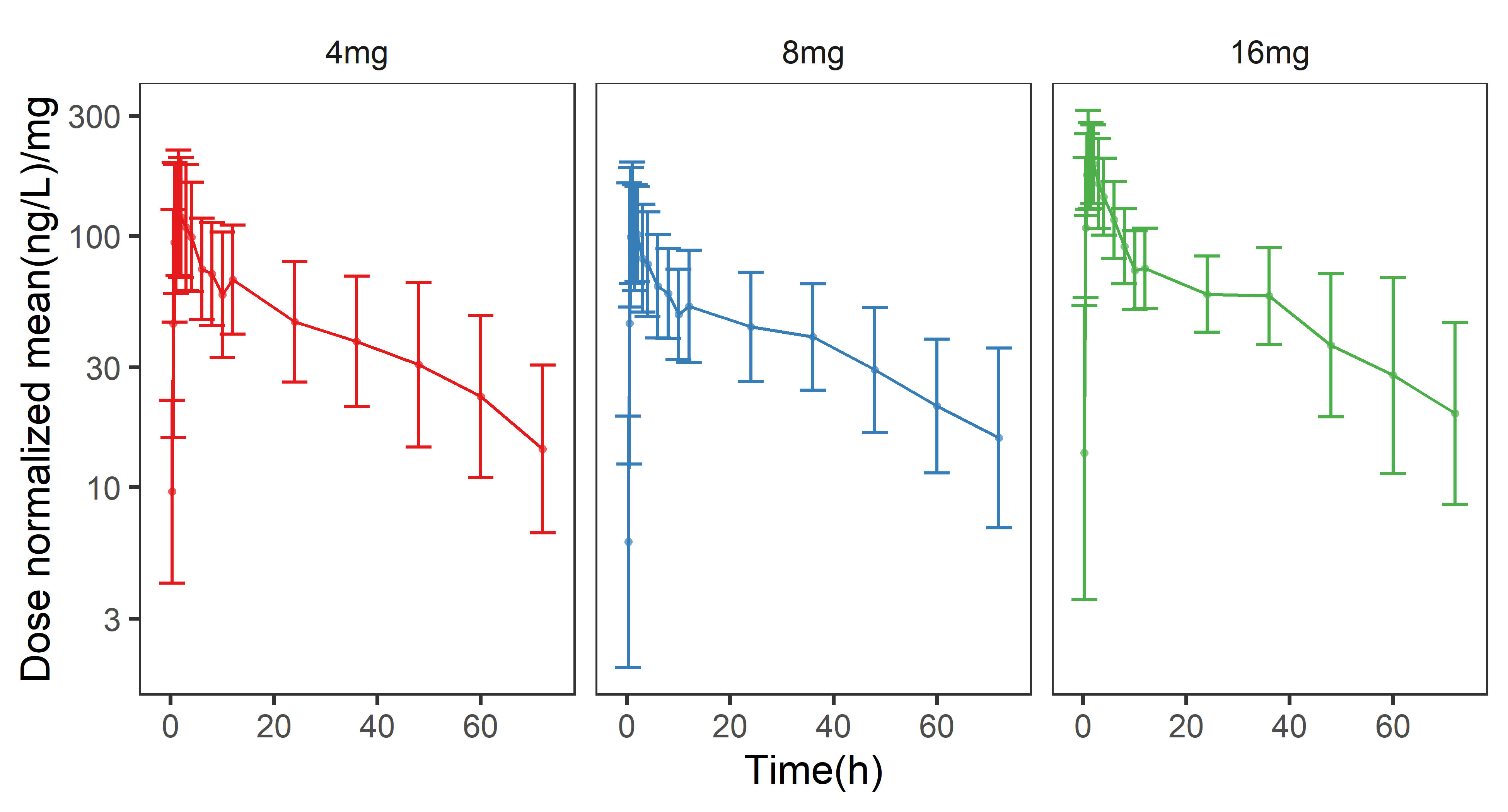


**Figure S2.** Dose normalized geometric mean of concentration-time graph for single dose of norbuprenorphine. The scale of y axis is log and of x is normal.


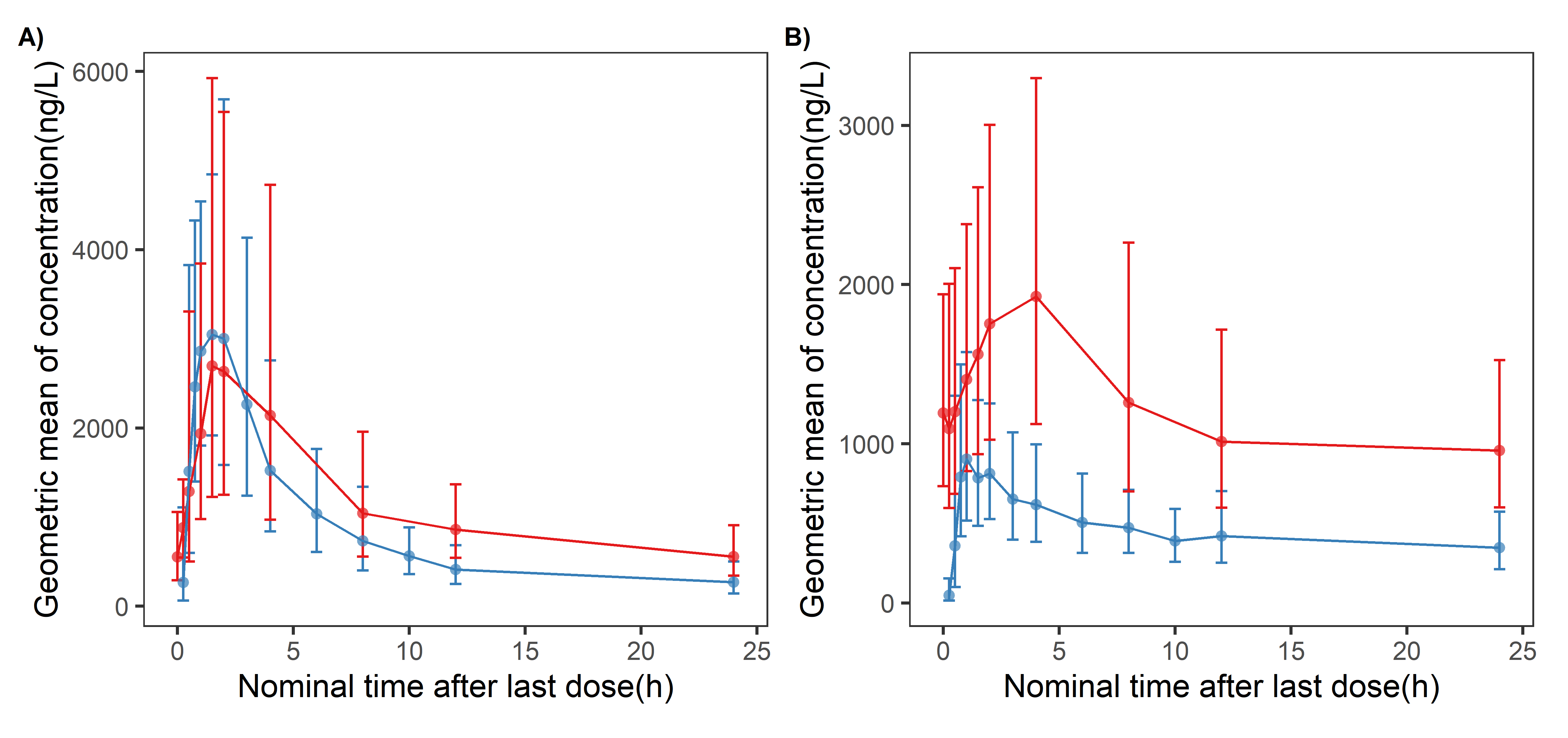


**Figure S3.** Comparing results between geometric mean of concentration after single/multiple doses and times ((A) buprenorphine; (B) norbuprenorphine). The red line is multiple dosing, and blue line indicates single dosing.

**
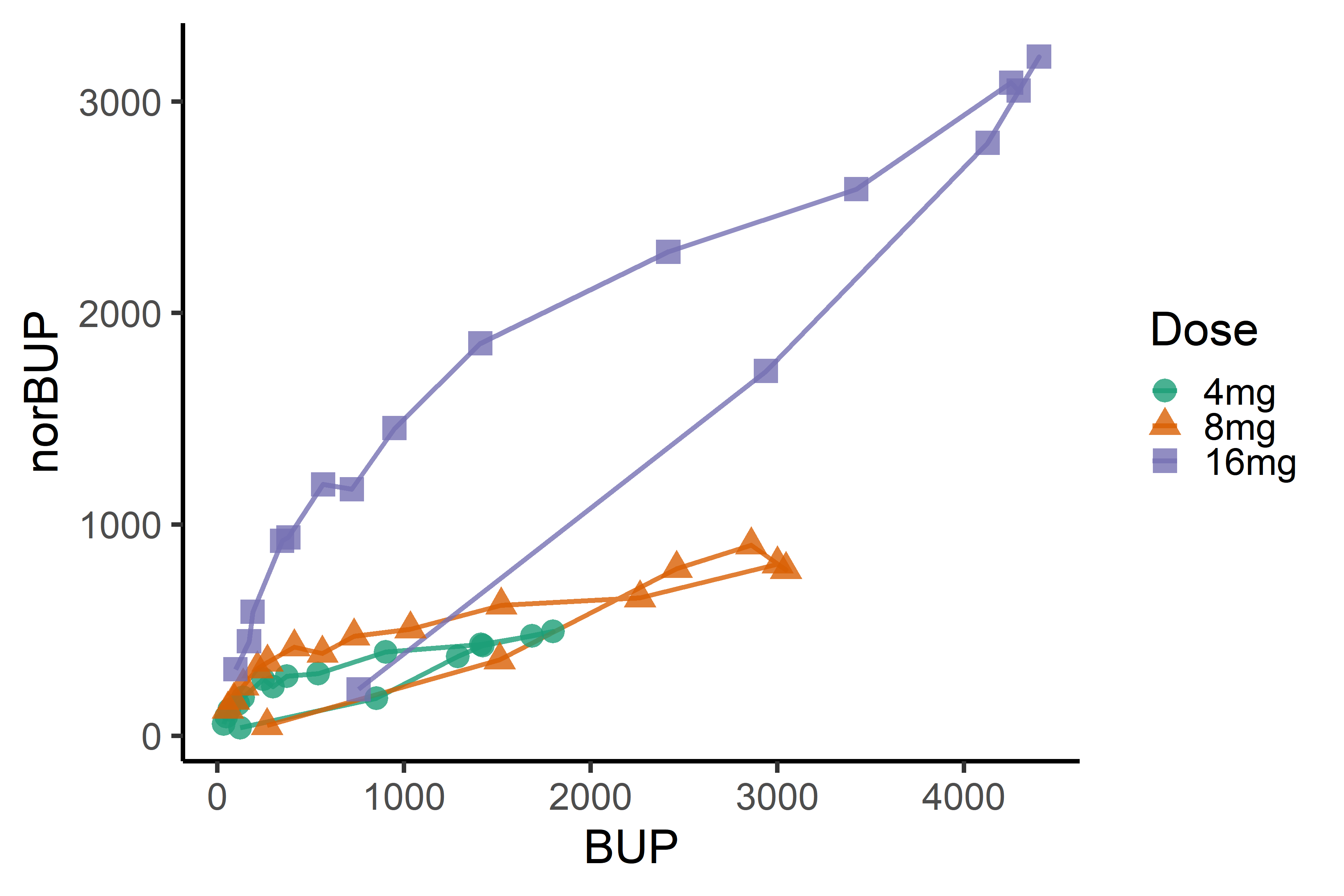
**

Figure S4. The hysteresis loop plot of buprenorphine and norbuprenorphine. The abscissa and ordinate coordinates for each point are the concentrations of buprenorphine and norbuprenorphine observed at the same time.


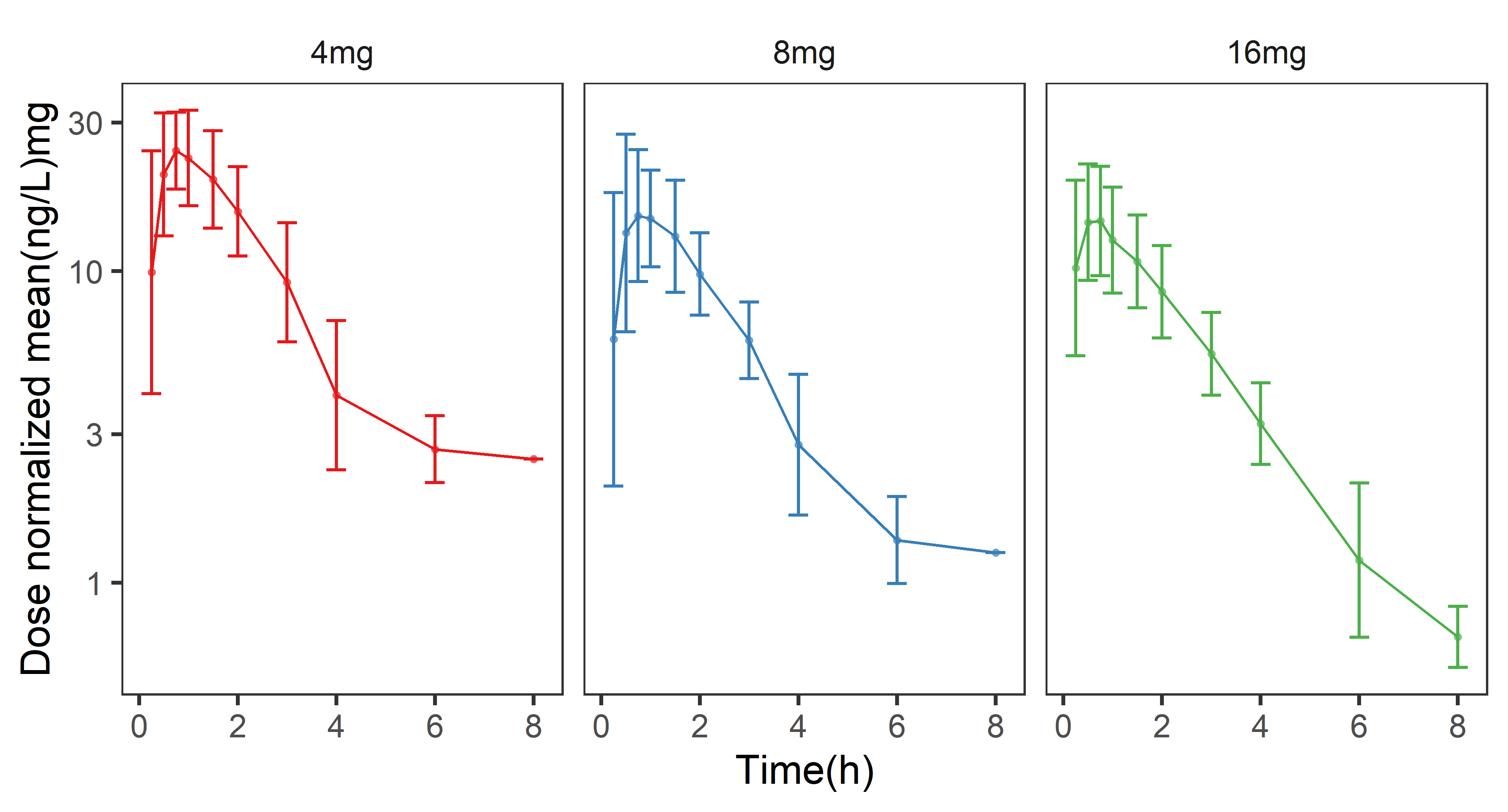


**Figure S5.** Dose normalized geometric mean of concentration-time graph for single dose of naloxone. The scale of y axis is log and of x is normal.


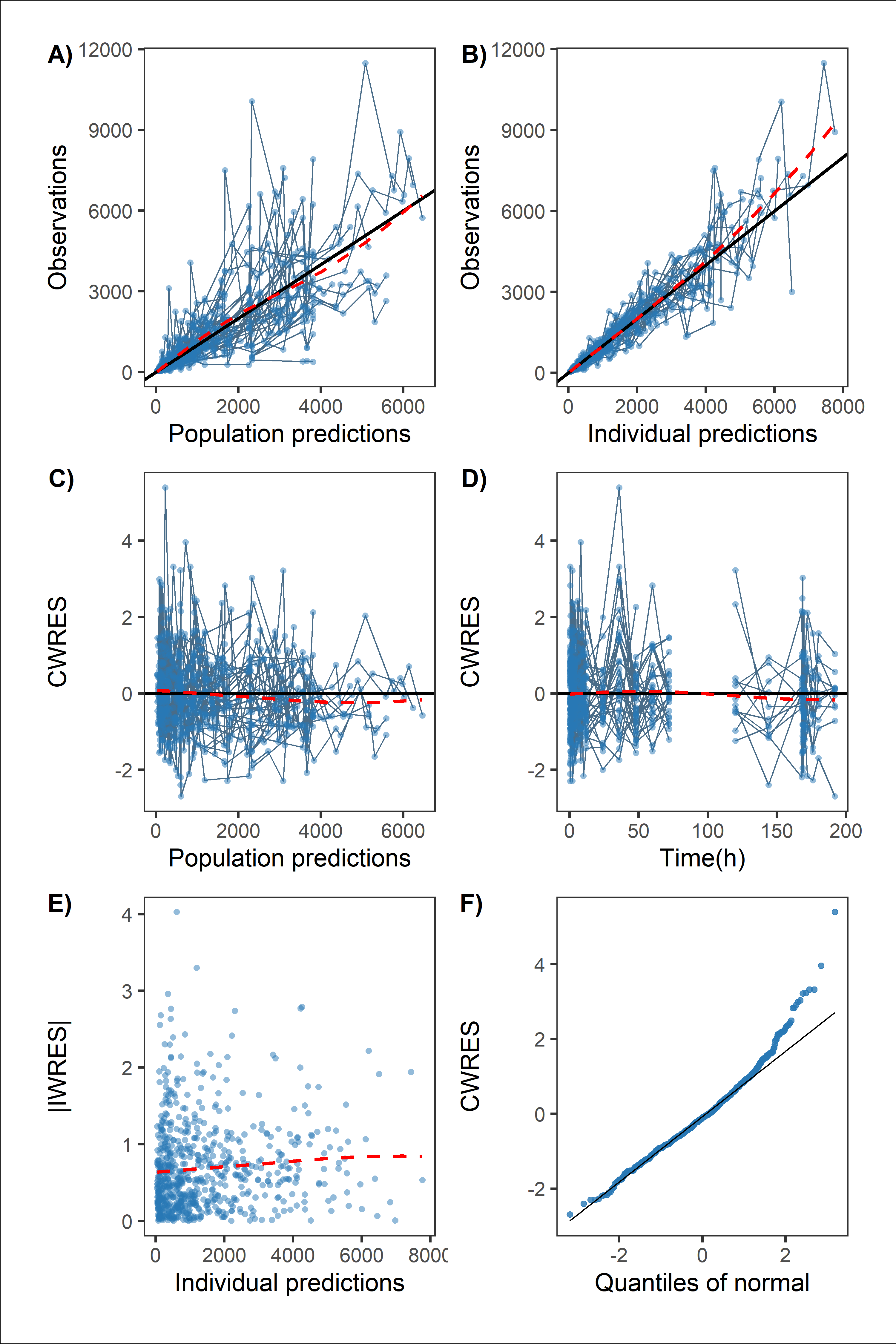


**Figure S6.** The goodness of fit plot for the final pharmacokinetic model of buprenorphine and norbuprenorphine to buprenorphine observation. The red line indicates the linear regression trendline and the black solid line means the reference line. Light blue circles represent observed data. (A) observations vs. population predictions; (B) observations vs. individual predictions; (C) Conditional weighted residuals (CWRES) vs. population predictions; (D) CWRES vs. time; (E) the absolute values of individual weighted residuals (|IWRES|) vs. individual prediction; (F) the quantile-quantile plot of CWRES.


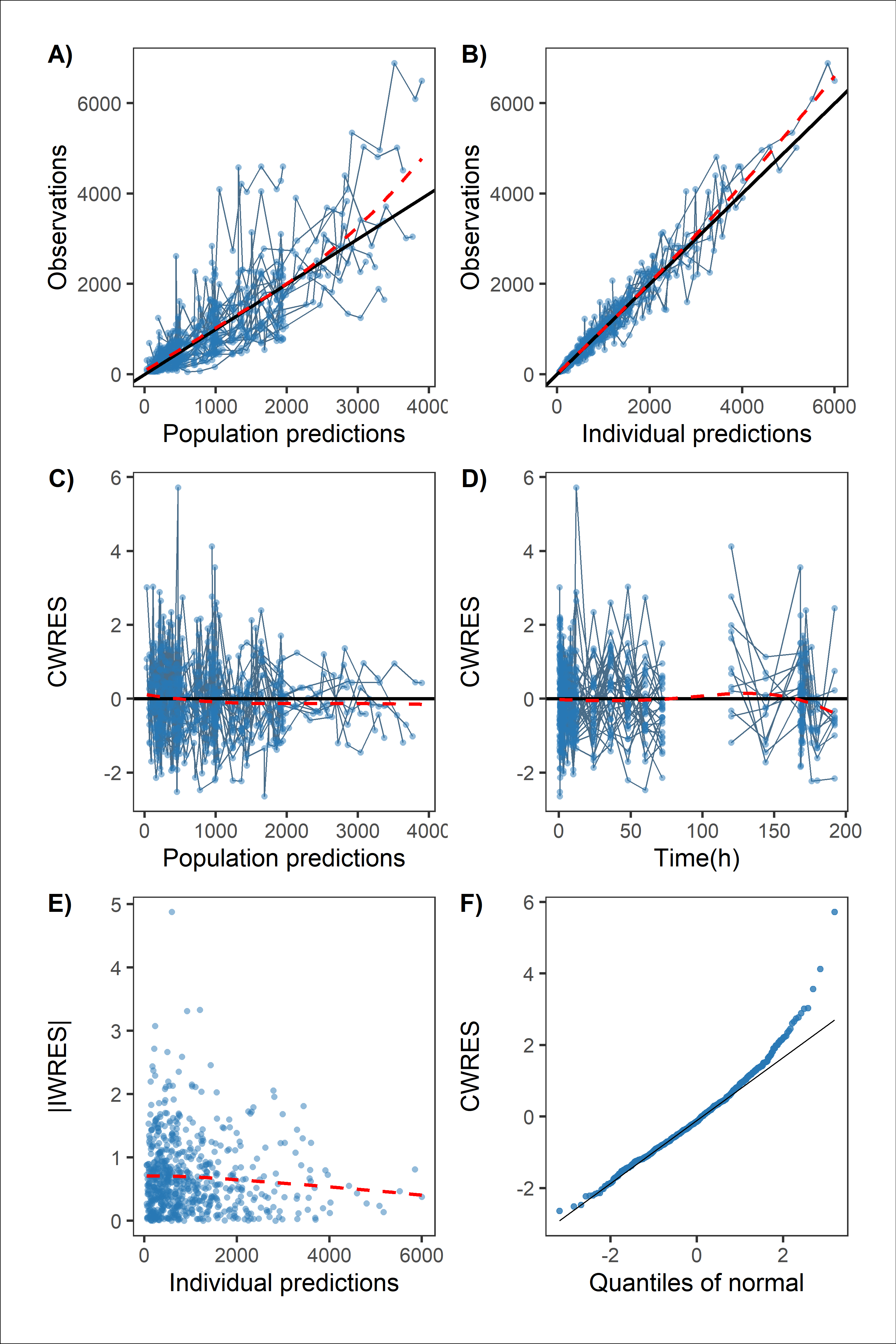


**Figure S7.** The goodness of fit plot for the final pharmacokinetic model of buprenorphine and norbuprenorphine to norbuprenorphine observation. The red line indicates the linear regression trendline and the black solid line means the reference line. Light blue circles represent observed data. (A) observations vs. population predictions; (B) observations vs. individual predictions; (C) Conditional weighted residuals (CWRES) vs. population predictions; (D) CWRES vs. time; (E) the absolute values of individual weighted residuals (|IWRES|) vs. individual prediction; (F) the quantile-quantile plot of CWRES.

**
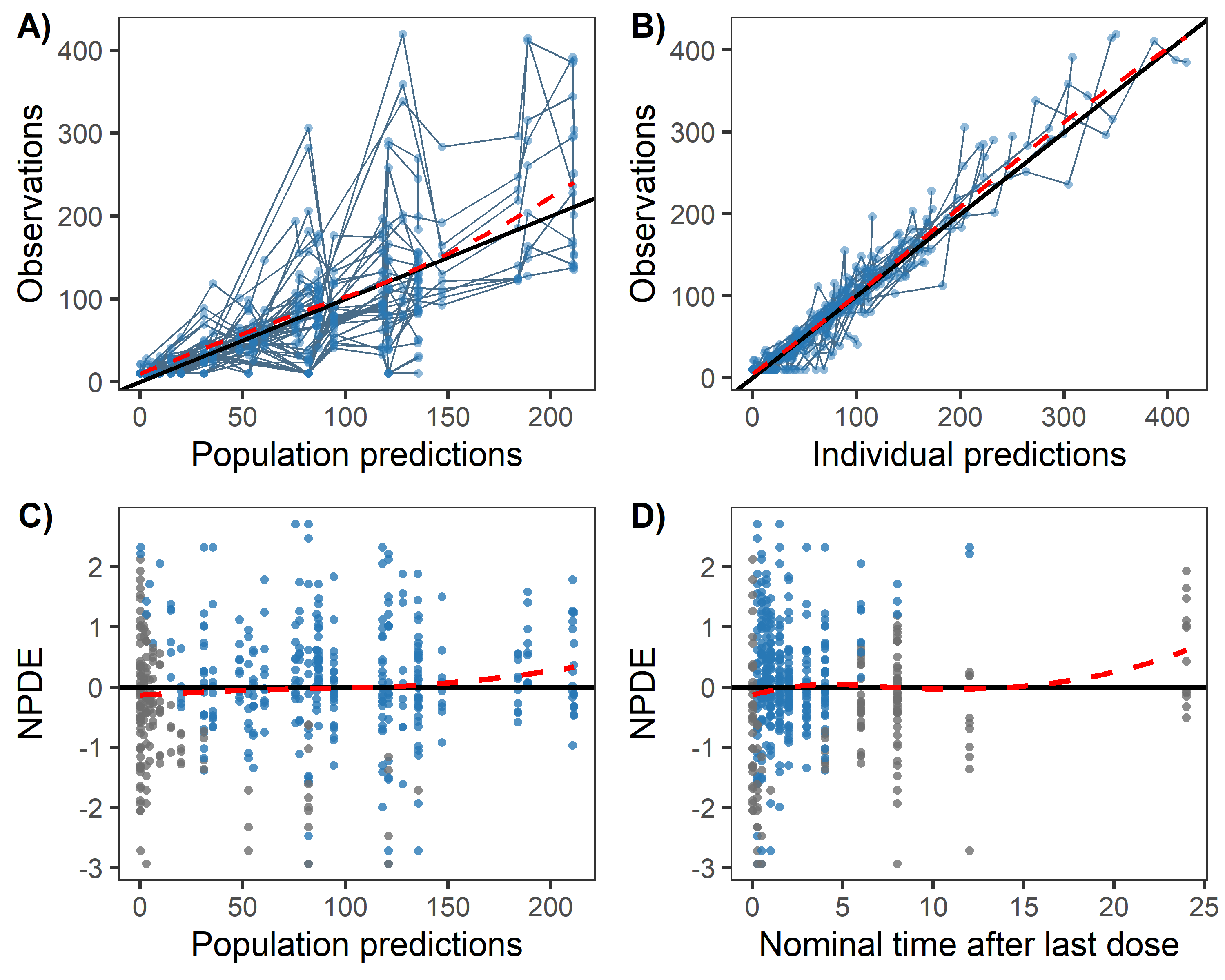
**

**Figure S8.** The goodness of fit plot for the final pharmacokinetic model of nloxone. The red line indicates the linear regression trendline and the black solid line means the reference line. Light blue circles represent observed data of single dose and gray circles represent observed data of repeat dose. (A) observations vs. population predictions; (B) observations vs. individual predictions; (C) Normalized prediction distribution error (NPDE) vs. population predictions; (D) Normalized prediction distribution error (NPDE) vs. nominal time after last dose.

**The code of final buprenorphine-norbuprenorphine model**

$PROB

$INPUT C ID TIME DV AMT CMT EVID MDV BQL DOSE NTALD TD TYPE AGE HI WT BMI NOS DIS

$DATA BUP.csv IGNORE=C IGNORE = (BQL.EQ.1)

$SUBROUTINES ADVAN13 TOL=9

$MODEL

COM (DEPOT, DEFDOS)

COM (CENT, DEFOBS)

COM(PERI)

COM(MET)

COM(MPERI)

$PK

TVCLP = THETA(1)

CLP = TVCLP*EXP(ETA(1))

TVV2 = THETA(2)

V2 = TVV2*EXP(ETA(2))

TVV3 = THETA(3)

V3 = TVV3*EXP(ETA(3))

TVKA = THETA(4)

KA = TVKA*EXP(ETA(4))

TVQP = THETA(5)

QP = TVQP*EXP(ETA(5))

TVN = THETA(6)

N = TVN*EXP(ETA(6))

TVMTT = THETA(7)

MTT = TVMTT*EXP(ETA(7))

KTR = (N+1)/MTT

TVSLDO=DOSE*(1+THETA(8)*(NOS-3))

SLDO = TVSLDO

FFACTOR = 1

IF(DOSE.EQ.16) FFACTOR = THETA(18)

TVFM = THETA(17)*FFACTOR

FM=TVFM

TVV4=THETA(9)

V4=TVV4*EXP(ETA(8))

TVCLM=THETA(10)

CLM=TVCLM*EXP(ETA(9))

TVV5=THETA(15)

V5=TVV5*EXP(ETA(10))

TVQM=THETA(16)

QM=TVQM*EXP(ETA(11))

LNFAC = LOG(2.5066)+(N+0.5)*LOG(N)-N

F1 = 0

F2 = 1

K23 = QP/V2

K32 = QP/V3

K24 = CLP/V2

K40 = CLM/V4

K45 = QM/V4

K54 = QM/V5

A_0(1) = 0

A_0(2) = 0

A_0(3) = 0

A_0(4) = 0

A_0(5) = 0

$DES

DADT(1) = EXP(LOG(SLDO+.00001)+LOG(KTR)+N*LOG(KTR*(T-TD)+.00001)-KTR*(T-TD)-LNFAC)-KA*A(1)

DADT(2) = KA*A(1) - K23*A(2) + K32*A(3) -K24*A(2)

DADT(3) = K23*A(2) - K32*A(3)

DADT(4) = FM*K24*A(2) - K40*A(4) -K45*A(4) + K54*A(5)

DADT(5) = K45*A(4) - K54*A(5)

$ERROR

CP = A(2)/V2*1000000

CM = A(4)/V4*1000000

IF(TYPE.EQ.2) THEN

IPRED = CP

W = SQRT(THETA(11)**2*IPRED**2 + THETA(12)**2)

Y = IPRED + W*EPS(1)

IRES = DV-IPRED

IWRES = IRES/W

ENDIF

IF(TYPE.EQ.3) THEN

IPRED = CM

W = SQRT(THETA(13)**2*IPRED**2 + THETA(14)**2)

Y = IPRED + W*EPS(2)

IRES = DV-IPRED

IWRES = IRES/W

ENDIF

$THETA

(0, 270);

(0, 377);

(0, 5870);

(0, 0.397);

(0, 404);

(8) FIX;

(0, 0.233);

(-0.035);

(0, 22.2);

(0, 264);

(0, 0.281);

(0) FIX;

(0, 0.22;

(0) FIX;

(0, 5170);

(0, 705);

(1) FIX; Fm of 4mg and 8mg

(1.98); Fm ratio of 16mg

$OMEGA

0.1;

1.84;

0.171;

0 FIX;

0.244;

0 FIX;

0.141;

1.43;

0.255;

0.203;

0.322;

$SIGMA

1 FIX; buprenorphine

1 FIX; norbuprenorphine

$EST METHOD=1 INTER MAXEVAL=99999 NOABORT SIG=2 POSTHOC

$COV PRINT=E MATRIX=S

**The code of final naloxone model**

$PROB

$INPUT C ID TIME DV AMT CMT EVID MDV BQL DOSE ADDL II NTALD TYPE AGE HI WT BMI NOS DIS

$DATA NLO.csv IGNORE=C

$SUBR ADVAN2 TRANS2

$PK

TVCL = THETA(1)

CL = TVCL*EXP(ETA(1))

TVV = THETA(2)

V = TVV*EXP(ETA(2))

TVKA = THETA(3)

KA = TVKA*EXP(ETA(3))

TVF1 = THETA(4)*(DOSE/8)**THETA(7)

F1 = TVF1

S2=V/1000000

$ERROR

IPRED = F

IF(COMACT==1) PREDV=IPRED

SD = SQRT(THETA(5)**2*IPRED**2 + THETA(6)**2)

LLOQ = 20

DEL= 1.0E-30

CUMD = PHI((LLOQ-IPRED)/SD)+DEL

IF (BQL.EQ.1) DV_LOQ=LLOQ

IF (BQL.EQ.0.OR.NPDE_MODE==1) THEN

F_FLAG=0

Y = IPRED + SD*EPS(1)

ENDIF

IF (BQL.EQ.1.AND.NPDE_MODE==0) THEN

F_FLAG=1

Y= CUMD

MDVRES=1

ENDIF

$THETA

(0,80);

(0,50);

(0,0.5);

0.01 FIX;

(0, 0.1);

(0, 3);

-0.3;

$OMEGA

0.1;

0.1;

0 FIX;

$SIGMA

1 FIX

$EST METHOD=1 LAPLACIAN INTERACTION NUMERICAL SLOW MAXEVALS=9999 NOABORT NSIG=3 SIGL=9 PRINT=10

$COV PRINT=E UNCONDITIONAL SLOW
