# Supplementary material for: Population pharmacokinetics of buprenorphine and naloxone sublingual combination in Chinese healthy volunteers and patients with opioid use disorder: Model-based dose optimization
Source: Front Pharmacol. 2023 Jan 19;14:1089862. doi: 10.3389/fphar.2023.1089862 (PMC9893638; doi:10.3389/fphar.2023.1089862)
Supplement: Supplementary file 2 [file Table2.DOCX]

Supplementary Material

**Sample processing**

**Buprenorphine**

Add 50 µL of 50 µg/L buprenorphine-D4 solution (internal standard) to a stoppered conical glass centrifuge tube, then then add 0.5 mL of plasma containing the drug and shake rapidly for 15 s. Subsequently, add 0.1 mL of aqueous sodium hydroxide solution at a concentration of 2 mol/L and shake rapidly for 15 s. Then add 3 mL of hexane for extracting and vortex for 1 min, then centrifuge under 3000 r/min for 10 min. Aspirate the supernatant in a centrifuge tube and dry in a vacuum at 45°C until dry. Add 200 µL of mobile phase solution to the centrifuge tube and shake rapidly for 15 s to dissolve the residue, then centrifuge for 10min under 3500r/min. Finally, 10 µL of supernatant was aspirated as sample for testing.

**Norbuprenorphine**

Add 50 µL of 50 µg/L norbuprenorphine-D3 solution (internal standard) to a stoppered conical glass centrifuge tube, then then add 0.5 mL of plasma containing the drug and shake rapidly for 15 s. Subsequently, add 0.1 mL of aqueous sodium hydroxide solution at a concentration of 2 mol/L and shake rapidly for 15 s. Then add 3 mL of ethyl acetate and dichloromethane (4:1) for extracting, and vortex for 1 min, then centrifuge under 3000 r/min for 10 min. Aspirate the supernatant in a centrifuge tube and dry in a vacuum at 45°C until dry. Add 100 µL of mobile phase solution to the centrifuge tube and shake rapidly for 15 s to dissolve the residue, then centrifuge for 10min under 3500r/min. Finally, 8 µL of supernatant was aspirated as sample for testing.

**Naloxone**

Add 50 µL of 8 µg/L naloxone-D5 solution (internal standard) to a stoppered conical glass centrifuge tube, then then add 0.5 mL of plasma containing the drug and shake rapidly for 15 s. Subsequently, add 0.1 mL of aqueous sodium hydroxide solution at a concentration of 2 mol/L and shake rapidly for 15 s. Then add 3 mL of ethyl acetate and dichloromethane (4:1) for extracting, and vortex for 1 min, then centrifuge under 3000 r/min for 10 min. Aspirate the supernatant in a centrifuge tube and dry in a vacuum at 45°C until dry. Add 100 µL of mobile phase solution to the centrifuge tube and shake rapidly for 15 s to dissolve the residue, then centrifuge for 10min under 3500r/min. Finally, 6 µL of supernatant was aspirated as sample for testing.

**Eluting program**

**Buprenorphine**

Chromatographic column: Agilent Eclipse plus C_18_ (4.6mm×150mm, 3.5μm); mobile phase: methanol-water (80:20, V: V, containing 0.005 mol/L ammonium formate and 0.1% formic acid); flow rate: 0.5mL/min; column temperature: 40°C; injection volume: 10 µL.

**Norbuprenorphine**

Chromatographic column: Agilent Eclipse plus C_18_ (4.6mm×150mm, 3.5μm); mobile phase: methanol-water (80:20, V: V, containing 0.005 mol/L ammonium formate and 0.1% formic acid); flow rate: 0.5mL/min; column temperature: 40°C; injection volume: 8 µL.

**Naloxone**

Chromatographic column: Agilent Eclipse plus C_18_ (4.6mm×150mm, 3.5μm); mobile phase: acetonitrile-water (36:64, V: V, containing 0.005 mol/L ammonium formate and 0.1% formic acid); flow rate: 0.5mL/min; column temperature: 40℃; injection volume: 6µL.

**Mass Spectrometry Conditions**

**Buprenorphine**

Electrospray ionization source, positive ionization, capillary voltage: 4000 V, spray gas pressure: 50 PSI, drying gas flow rate: 10 L/min, drying gas temperature: 350°C. The scanning mode was selective ion monitoring, buprenorphine m/z 468.3→55.2, collision energy: 58 V, cleavage voltage: 210 V, quadrupole capture time: 0.3 s, electron multiplier: 400 V; internal standard buprenorphine-D4 m/z 472.3→59.2, collision energy: 58 V, cleavage voltage: 210 V, quadrupole capture time: 0.1 s, electron multiplier: 400 V.

**Norbuprenorphine**

Electrospray ionization source, positive ionization, capillary voltage: 4000 V, spray gas pressure: 50 PSI, drying gas flow rate: 10 L/min, drying gas temperature: 350°C. Scanning mode was selective ion monitoring, norbuprenorphine m/z 414.4→101.4, collision energy: 38 V, cleavage voltage: 200 V, quadrupole capture time 0.3 s, electron multiplier 500 V; internal standard norbuprenorphine-D3 m/z 417.3→101.3, collision energy: 40 V, cleavage voltage: 200 V, quadrupole capture time: 0.1 s, electron multiplier: 500 V.

**Naloxone**

Electrospray ionization source, positive ionization, capillary voltage: 4000V, spray gas pressure: 50PSI, drying gas flow rate: 10L/min, drying gas temperature: 350°C. Scanning mode was selective ion monitoring, naloxone m/z 328.2→310.2, collision energy: 18V, cleavage voltage: 120V, quadrupole capture time: 0.3s, electron multiplier: 400V; internal standard naloxone-D5 m/z 333.3→315.3, collision energy: 15V, cleavage voltage: 120V, quadrupole capture time: 0.2s, electron multiplier: 400V.

**Standard curve preparation**

**Buprenorphine**

Take blank tubes and add 50 μL of buprenorphine control solution at concentrations of 500, 1000, 5000, 10000, 50000, 100000 and 150000 ng/L, respectively. After evaporation, 0.5 mL blank plasma was added to each tube and shaken for 15 s, so that the plasma drug concentrations were equivalent to 50, 100, 500, 1000, 5000, 10000 and 15000 ng/L, respectively, and processed according to the sample treatment method. The ratio of buprenorphine peak area to internal standard peak area ($y$) was used as the vertical coordinate, and the ratio of plasma buprenorphine concentration to internal standard concentration ($x$) was used as the horizontal coordinate with a weighting factor of 1/x^2^. By HPLC-MS/MS analysis, the plasma buprenorphine concentrations showed good linearity in the range of 50-15000 ng/L, and the typical representative equation was: $y=1.0013*x-8.8757*{10}^{-4}$, R^2^=0.9982 (n=7).

**Norbuprenorphine**

Take blank tubes and add 50 μL of norbuprenorphine control solution at concentrations of 500, 1000, 5000, 10000, 50000, 100000 and 150000 ng/L, respectively. After evaporation, 0.5 mL blank plasma was added to each tube and shaken for 15 s, so that the plasma drug concentrations were equivalent to 50, 100, 500, 1000, 5000, 10000 and 15000 ng/L, respectively, and processed according to the sample treatment method. The ratio of norbuprenorphine peak area to internal standard peak area ($y$) was used as the vertical coordinate, and the ratio of plasma norbuprenorphine concentration to internal standard concentration ($x$) was used as the horizontal coordinate with a weighting factor of 1/x^2^. By HPLC-MS/MS analysis, the plasma norbuprenorphine concentrations showed good linearity in the range of 50-10000 ng/L, and the typical representative equation was: $y=1.2152*x-0.0017$, R^2^=0.9963 (n=7).

**Naloxone**

Take blank tubes and add 50 μL of naloxone control solution at concentrations of 200, 500, 1000, 2000, 5000, 10000, 15000 ng/L, respectively. After evaporation, 0.5 mL blank plasma was added to each tube and shaken for 15 s, so that the plasma drug concentrations were equivalent to 20, 50, 100, 200, 500, 1000, and 1500 ng/L, respectively, and processed according to the sample treatment method. The ratio of naloxone peak area to internal standard peak area ($y$) was used as the vertical coordinate, and the ratio of plasma naloxone concentration to internal standard concentration ($x$) was used as the horizontal coordinate with a weighting factor of 1/x^2^. By HPLC-MS/MS analysis, the plasma naloxone concentrations showed good linearity in the range of 20-1500 ng/L, and the typical representative equation was: $y=1.2159*x-0.0023$, R^2^=0.9965 (n=7).

**Precision, accuracy, and extraction recovery test**

**Buprenorphine**

According to the preparation method of the standard curve, 15 portions buprenorphine plasma solutions of low (100 ng/L), medium (1000 ng/L) and high (10000 ng/L) concentrations were prepared respectively. The prepared solutions were divided into 3 batches of 5 portions each, and the samples were injected at the same time together with the standard curve of each batch according to the sample processing method. The concentrations of the quality control samples were calculated and compared with the prepared concentrations to obtain the accuracy and precision of this measurement method. According to the meaning of extraction recovery of analytes, the peak areas of buprenorphine and buprenorphine-D4 recovered from plasma were divided by the peak areas of the corresponding concentrations of standard solutions as extraction recoveries. The results showed that the extraction recoveries of buprenorphine were 72.57%, 66.50%, 64.70% and 64.42% for low (100 ng/L), medium (1000 ng/L), high (10000 ng/L) and internal standards, respectively. Thus, the extraction recoveries of the three concentrations of the drug, low, medium, and high, had basically the same degree of influence on the results of the assay. The extraction recoveries and precision are shown in Table SS1 and Table SS2, respectively.

**Norbuprenorphine**

According to the preparation method of the standard curve, 15 portions norbuprenorphine plasma solutions of low (100 ng/L), medium (1000 ng/L) and high (8000 ng/L) concentrations were prepared respectively. The prepared solutions were divided into 3 batches of 5 portions each, and the samples were injected at the same time together with the standard curve of each batch according to the sample processing method. The concentrations of the quality control samples were calculated and compared with the prepared concentrations to obtain the accuracy and precision of this measurement method. According to the meaning of extraction recovery of analytes, the peak areas of norbuprenorphine and norbuprenorphine-D3 recovered from plasma were divided by the peak areas of the corresponding concentrations of standard solutions as extraction recoveries. The results showed that the extraction recoveries of norbuprenorphine were 68.21%, 68.23%, 66.07% and 66.15% for low (100 ng/L), medium (1000 ng/L), high (8000 ng/L) and internal standards, respectively. Thus, the extraction recoveries of the three concentrations of the drug, low, medium, and high, had basically the same degree of influence on the results of the assay. The extraction recoveries and precision are shown in Table SS1 and Table SS2, respectively.

**Naloxone**

According to the preparation method of the standard curve, 15 portions naloxone plasma solutions of low (50 ng/L), medium (200 ng/L) and high (1000 ng/L) concentrations were prepared respectively. The prepared solutions were divided into 3 batches of 5 portions each, and the samples were injected at the same time together with the standard curve of each batch according to the sample processing method. The concentrations of the quality control samples were calculated and compared with the prepared concentrations to obtain the accuracy and precision of this measurement method. According to the meaning of extraction recovery of analytes, the peak areas of naloxone and naloxone-D5 recovered from plasma were divided by the peak areas of the corresponding concentrations of standard solutions as extraction recoveries. The results showed that the extraction recoveries of naloxone were 81.10%, 85.97%, 81.89% and 81.14% for low (50 ng/L), medium (200 ng/L), high (1000 ng/L) and internal standards, respectively. Thus, the extraction recoveries of the three concentrations of the drug, low, medium, and high, had basically the same degree of influence on the results of the assay. The extraction recoveries and precision are shown in Table SS1 and Table SS2, respectively.

Table SS1. The extraction recoveries of buprenorphine, norbuprenorphine, naloxone and internal standard in plasma.

| Concentration (ng/L) | **Buprenorphine** | | | **Norbuprenorphine** | | | **Naloxone** | | |
| --- | --- | --- | --- | --- | --- | --- | --- | --- | --- |
|  | Mean | SD | RSD (%) | Mean | SD | RSD (%) | Mean | SD | RSD (%) |
| 100 | 72.57 | 5.11 | 7.04 | 68.21 | 7.59 | 11.13 | 81.10 | 5.22 | 6.44 |
| 1000 | 66.50 | 6.54 | 9.83 | 68.23 | 6.75 | 9.89 | 85.97 | 7.85 | 9.13 |
| 10000 | 64.70 | 4.24 | 6.55 | 66.07 | 6.38 | 9.66 | 81.89 | 5.52 | 6.74 |
| 5000  (internal standard) | 64.42 | 4.01 | 6.23 | 66.15 | 6.83 | 10.32 | 81.14 | 5.30 | 6.53 |

*SD, standard deviation; RSD, relative standard deviation.

Table SS2. Results of accuracy and precision of buprenorphine, norbuprenorphine, and naloxone in plasma.

| Number of samples | **Buprenorphine** | | | **Norbuprenorphine** | | | **Naloxone** | | |
| --- | --- | --- | --- | --- | --- | --- | --- | --- | --- |
|  | 15 | 15 | 15 | 15 | 15 | 15 | 15 | 15 | 15 |
| Mean (ng/L) | 97.73 | 963.29 | 10085.71 | 99.26 | 981.94 | 7931.28 | 50.10 | 191.57 | 984.56 |
| SD | 3.06 | 35.90 | 316.26 | 9.02 | 39.40 | 301.46 | 2.13 | 6.15 | 26.95 |
| Within-batch precision (%) | 3.38 | 4.03 | 3.39 | 9.82 | 4.33 | 4.11 | 4.60 | 3.47 | 2.96 |
| Between-run precision (%) | 2.98 | 3.49 | 7.84 | 9.27 | 5.90 | 8.49 | 6.52 | 6.63 | 6.92 |
| Deviation (%) | -2.27 | -3.67 | 0.86 | -0.74 | -1.81 | -0.86 | 0.21 | -4.21 | -1.54 |
| Accuracy (%) | 97.73 | 96.33 | 100.86 | 99.26 | 98.19 | 99.14 | 100.21 | 95.79 | 98.46 |

*SD, standard deviation.
